# Betamethasone improved near‐term neonatal lamb lung maturation in experimental maternal asthma

**DOI:** 10.1113/EP091997

**Published:** 2024-10-22

**Authors:** Joshua L. Robinson, Andrea J. Roff, Sarah J. Hammond, Jack R. T. Darby, Ashley S. Meakin, Stacey L. Holman, Andrew Tai, Tim J. M. Moss, Catherine G. Dimasi, Sarah M. Jesse, Michael D. Wiese, Andrew N. Davies, Beverly S. Muhlhausler, Robert J. Bischof, Megan J. Wallace, Vicki L. Clifton, Janna L. Morrison, Michael J. Stark, Kathryn L. Gatford

**Affiliations:** ^1^ Robinson Research Institute University of Adelaide Adelaide South Australia Australia; ^2^ Adelaide Medical School University of Adelaide Adelaide South Australia Australia; ^3^ Early Origins of Adult Health Research Group, Health and Biomedical Innovation, Clinical and Health Sciences University of South Australia Adelaide South Australia Australia; ^4^ School of Biomedicine University of Adelaide Adelaide South Australia Australia; ^5^ Respiratory and Sleep Medicine Women's & Children's Hospital North Adelaide South Australia Australia; ^6^ Department of Obstetrics and Gynaecology Monash University Clayton Victoria Australia; ^7^ Centre for Pharmaceutical Innovation, Clinical & Health Sciences University of South Australia Adelaide South Australia Australia; ^8^ Biomedicine Discovery Institute Monash University Frankston Victoria Australia; ^9^ Health and Biosecurity Commonwealth Scientific and Industrial Research Organisation Adelaide South Australia Australia; ^10^ Institute of Innovation, Science, and Sustainability Federation University Australia Berwick Victoria Australia; ^11^ The Ritchie Centre Hudson Institute of Medical Research Clayton Victoria Australia; ^12^ Mater Medical Research Institute University of Queensland South Brisbane Queensland Australia; ^13^ Department of Neonatal Medicine Women's & Children's Hospital North Adelaide South Australia Australia

**Keywords:** animal, disease models, glucocorticoids, newborn, physiopathology, pregnancy complications, respiratory distress syndrome, sheep

## Abstract

Maternal asthma is associated with increased rates of neonatal lung disease, and fetuses from asthmatic ewes have fewer surfactant‐producing cells and lower surfactant‐protein B gene (*SFTPB*) expression than controls. Antenatal betamethasone increases lung surfactant production in preterm babies, and we therefore tested this therapy in experimental maternal asthma. Ewes were sensitised to house dust mite allergen, and an asthmatic phenotype induced by fortnightly allergen lung challenges; controls received saline. Pregnant asthmatic ewes were randomised to receive antenatal saline (asthma) or 12 mg intramuscular betamethasone (asthma+beta) at 138 and 139 days of gestation (term = 150 days). Lambs were delivered by Caesarean section at 140 days of gestation and ventilated for 45 min before tissue collection. Lung function and structure were similar in control lambs (*n* = 16, 11 ewes) and lambs from asthma ewes (*n* = 14, 9 ewes). Dynamic lung compliance was higher in lambs from asthma+beta ewes (*n* = 12, 8 ewes) compared to those from controls (*P* = 0.003) or asthma ewes (*P* = 0.008). Lung expression of surfactant protein genes *SFTPA* (*P* = 0.048) and *SFTPB* (*P* < 0.001), but not *SFTPC* (*P* = 0.177) or *SFTPD* (*P* = 0.285), was higher in lambs from asthma+beta than those from asthma ewes. Female lambs had higher tidal volume (*P* = 0.007), dynamic lung compliance (*P* < 0.001), and *SFTPA* (*P* = 0.037) and *SFTPB* gene expression (*P* = 0.030) than males. These data suggest that betamethasone stimulates lung maturation and function of near‐term neonates, even in the absence of impairment by maternal asthma.

## Abstract (highlights)

- **What is the central question of this study?**
  Does antenatal betamethasone before near‐term delivery mitigate the increased risk of neonatal lung disease in a sheep model of maternal asthma?
- **What is the main finding and its importance?**
  Maternal asthma did not impair neonatal lung function or reduce surfactant gene expression, likely reflecting a mild maternal and fetal phenotype in this cohort. Nevertheless, antenatal maternal betamethasone treatment in asthmatic pregnancies increased dynamic lung compliance and surfactant protein A and B mRNA expression in newborn lambs. These data suggest potential benefits of betamethasone on lung maturation when mothers have asthma.

## 1 INTRODUCTION

Asthma is a chronic condition characterised by hyperresponsiveness and inflammation of the airways that affects ∼9–17% of pregnant women worldwide (Das et al., 2021; Fujino et al., 2021; Kwon et al., 2006). Approximately 50% of pregnant women with asthma experience loss of control, involving increased symptom severity, or asthma exacerbations, leading to a need for medical intervention and increased medication use (Grzeskowiak et al., 2016). Maternal asthma in pregnancy is associated with increased risks of complications including gestational diabetes, preeclampsia, preterm delivery and Caesarean section (Hodyl et al., 2014; Murphy et al., 2011; Robinson, Gatford, Hurst, et al., 2023; Wang et al., 2014). Maternal asthma is also associated with increased risks of adverse neonatal outcomes including perinatal mortality, nursery admission, transient tachypnoea of the newborn (TTN), respiratory distress syndrome (RDS), low birthweight and small for gestational age (Hodyl et al., 2014; Mendola et al., 2014; Murphy et al., 2011, 2013; Robinson, Gatford, Hurst, et al., 2023), but the mechanisms are largely unknown. RDS and TTN are both associated with increased work of breathing and insufficient alveolar surfactant (Machado et al., 2011; McGillick et al., 2017; Reuter et al., 2014). In an experimental sheep model of maternal asthma before and during pregnancy, the lungs of near‐term fetuses from asthmatic ewes had fewer surfactant‐producing type II alveolar epithelial cells and lower surfactant protein gene expression compared to controls (Clifton et al., 2016; Wooldridge et al., 2019), suggesting that maternal asthma leads to newborn surfactant‐deficiency.

Antenatal corticosteroids (ACS) mature the fetal lung by increasing numbers of type II alveolar epithelial cells, increasing surfactant production, and thinning the alveolar walls (Polglase et al., 2007; Wallace et al., 1995, 1996). Treatment with ACS is recommended for women at risk of preterm delivery before 34–37 weeks’ completed gestation, depending on jurisdiction: 24 mg given in two (betamethasone) or four (dexamethasone) doses over the 48 h prior to expected delivery (Antenatal Corticosteroid Clinical Practice Guidelines Panel, 2015; Reddy et al., 2021; World Health Organization, 2022). ACS is not currently recommended for use before term delivery. However, given the evidence of lung immaturity in near‐term fetal sheep from asthmatic ewes (Clifton et al., 2016; Wooldridge et al., 2019) and increased risk of RDS in preterm and term human neonates exposed to maternal asthma (Mendola et al., 2014), we hypothesised that ACS therapy in pregnancies complicated by asthma would reduce the incidence of lung morbidities at term birth. We, therefore, compared neonatal lung function, lung structure, and surfactant protein gene expression in lambs from control ewes, asthmatic ewes treated with saline (asthma), and asthmatic ewes treated with antenatal betamethasone (asthma+beta). We did not use a control group treated with antenatal betamethasone given that steroids are not clinically recommended for women who are expected to deliver at term (World Health Organization, 2022); we only explored betamethasone as a potential intervention for the offspring of asthmatic mothers. We also explored the effect of lamb sex on outcomes and whether responses to treatment differed between sexes.

## 2 METHODS

### 2.1 Ethics approval

The South Australian Health and Medical Research Institute (SAHMRI) Animal Ethics Committee approved this study (SAM455.19) according to the Australian code for the care and use of animals for scientific purposes (National Health & Medical Research Council, 2013). All investigators adhered to the ethical principles outlined by Grundy (2015) and the principles of the 3Rs (Tannenbaum & Bennett, 2015).

### 2.2 Animals and induction of maternal asthma phenotype

**FIGURE 1:**
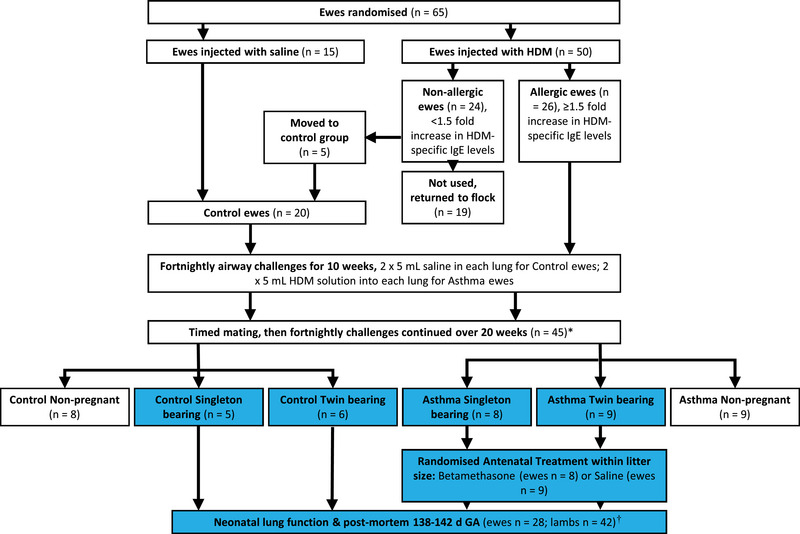
Flow diagram with ewe and lamb numbers. Pregnant ewes included in this study are indicated in blue boxes. HDM, house dust mite; HDM‐specific IgE, house dust mite‐specific immunoglobulin E. *Ewe death (*n* = 1 control). †In utero lamb death (*n* = 1) unrelated to experimental procedures.

Ewes were sensitised to house dust mite (HDM) and then subjected to repeated airway challenges with HDM to induce an asthmatic phenotype (Figure 1), following our published protocols (Bischof et al., 2003; Clifton et al., 2016). Control ewes received saline airway challenges. Ewe lung function was measured in late pregnancy (∼132 days of gestation, dG), immediately prior to challenge with HDM. Ewe lung eosinophils were measured in bronchoalveolar lavage collected before and 48 h after this challenge. Further details on sensitisation, induction of asthma and phenotype measures are provided in Supporting information, Supplementary methods.

### 2.3 Mating, treatments, lamb delivery and ventilation

After 10 weeks of HDM challenges, ewes were fitted with an intravaginal controlled internal drug release device containing progesterone (Eazi‐breed CIDR, Zoetis Australia Pty Ltd, Rhodes, NSW, Australia) to synchronise oestrus prior to mating with Merino rams. Fortnightly airway challenges were continued throughout the study. Pregnancy was confirmed by ultrasound at 55 dG (Figure 1). Singleton and twin‐bearing asthmatic ewes were randomised using a random number generator to receive saline or betamethasone (Celestone Chronodose 11.6 mg, Schering Plough, Baulkham Hills, NSW, Australia), with intramuscular injections 48 and 24 h prior to delivery, consistent with clinical practice (Antenatal Corticosteroid Clinical Practice Guidelines Panel, 2015). Control ewes received saline injections. A 150 mg dose of medroxyprogesterone acetate was given intramuscularly to all ewes 6–9 days prior to antenatal injections to prevent premature labour due to betamethasone‐induced progesterone withdrawal (Jenkin et al., 1985; Jobe et al., 2003). Lambs were delivered by Caesarean section at 140 dG and ventilated for 45 min with a volume guarantee strategy, as detailed in Supplementary methods. After ventilation, the lambs were humanely killed with sodium pentobarbitone (20 mg/kg, Virbac Australia, Peakhurst, NSW, Australia). The left lung was removed, weighed, and inflation fixed (20 cmH_2_0) in 4% paraformaldehyde. Samples from the fixed left cranial and left caudal lobes of each lung were paraffin‐embedded and sectioned (4 µm), and sections from the right caudal lobe were frozen in liquid nitrogen (Westover et al., 2012). Additional lamb tissues were extensively sampled consistent with best practice (Morrison et al., 2018).

### 2.4 Neonatal lung maturation

Tissue‐to‐airspace ratio and type II alveolar epithelial cell density were determined in caudal and cranial lung sections from each lamb (Lock et al., 2015). Surfactant protein gene expression was determined by quantitative real‐time PCR (Lock et al., 2017; McGillick et al., 2013; Orgeig et al., 2010). Concentrations of glucocorticoids (cortisol, cortisone, 11‐deoxycortisol and corticosterone) in arterial plasma prior to delivery and in lung tissue collected at the end of the study were determined by mass spectrophotometry (Dimasi et al., 2024; Lock et al., 2023). These analyses are described in more detail in the Supplementary methods.

### 2.5 Statistical analyses

Maternal lung function and immune responses to treatment, measured before allocating asthmatic ewes to saline or betamethasone treatment, were compared between control and asthmatic ewes by one‐way ANOVA. Repeated measures ANOVA was used to compare the proportion of eosinophils in bronchoalveolar lavage (BAL) before and after the airway challenge. One fetus (control female) died in utero, resulting in the delivery of a total of 42 lambs for ventilation and tissue collection. Four lambs (1 control male, 1 asthma male, 1 asthma female and 1 asthma+beta male) were excluded from functional analyses due to changes in the position and ventilation strategy during the initial optimisation of the protocol. Four lambs (1 control male, 2 asthma females and 1 asthma+beta female) were excluded from tissue‐to‐airspace ratio analyses due to poor fixation of the lung. Any missing blood gas or ventilation data for the remaining animals was due to machine calibrations. Lamb glucocorticoid and lung structural outcomes were analysed using treatment and lamb sex as factors, plus lung region as a repeated measure, with ewe as a random factor to correct for the effects of maternal environment. Singleton (*n* = 13) and twin (*n* = 15) pregnancies were included resulting in 43 lambs. In preliminary analyses, litter size had minimal effect on outcomes, and, therefore, we prioritised lamb sex for inclusion in statistical analysis; there were insufficient animal numbers to include both factors. Data collected during the lamb ventilation protocol were analysed by a linear mixed model using treatment and lamb sex as factors, plus time as a repeated measure, and ewe as a random factor to correct for the effects of the maternal environment. A Bonferroni correction was used to compare groups where there was an overall effect of ewe treatment. Where interactions were significant, further subgroup analyses were performed. Data were analysed using SPSS version 27 (IBM Corp., Armonk, NY, USA). If *P* < 0.05 the null hypothesis was rejected. Data are presented as means (standard deviation, SD).

## 3 RESULTS

### 3.1 Ewe and lamb phenotype

In late pregnancy (∼132 dG) there was no difference in pre‐challenge dynamic lung compliance between saline (0.0367 [0.0130] L/cmH_2_O) and asthma ewes (0.0333 [0.0140] L/cmH_2_O; *P* = 0.529). Although we did not see an effect of asthma on lung function within the pregnant animals included in the present paper, dynamic lung compliance decreased more in asthmatic than control ewes between premating and ∼132 dG in analyses of the whole cohort including non‐pregnant ewes (*P* = 0.040). Eosinophil concentrations in BAL were not different between control and asthma ewes either pre‐ (12,690 [18,957] n/mL cf. 6770 [10,319] n/mL; *P* = 0.294) or post‐challenge (91,486 [279,191] cf. 26,808 [53,339] n/mL; *P* = 0.357). Eosinophil concentrations were higher 48 h post‐challenge than pre‐challenge in both control (*P = *0.045) and asthma (*P *= 0.008) groups.

At 140 dG, there was an overall effect of treatment on ewe weight (*P *= 0.037, Table 1) but in pairwise comparisons, body weight was not different between control ewes and asthma (*P* = 0.164) or asthma+beta ewes (*P* = 0.051), nor between asthma and asthma+beta ewes (*P *= 1.000). When comparing all groups, treatment and lamb sex did not affect absolute or relative lamb body weight at delivery, nor absolute or relative weights of lamb lungs or brains (Table 1). In additional analyses combining all lambs from asthmatic ewes, absolute birth weight did not differ between lambs from control and asthmatic ewes (*P* = 0.523), but birth weight relative to maternal weight was 12.5% lower in lambs of asthmatic ewes compared to those born to control ewes (*P* = 0.046).

**TABLE 1:** Ewe and lamb weights at delivery^a^.

	Control		Asthma		Asthma+beta		Significance		
	Male	Female	Male	Female	Male	Female	*P*_treatment_	*P*_lamb sex_	*P*_interaction_
Number of ewes	11		9		8				
Ewe body weight (kg)	73.3 (6.8)		78.9 (4.6)		80.7 (6.7)		**0.037** ^c^		
Number of lambs (numbers per litter size)^b^	9 (4 S, 5 T)	8 (1 S, 7 T)	8 (1 S, 7 T)	6 (3 S, 3 T)	5 (2 S, 3 T)	7 (2 S, 5 T)			
Lamb birth weight (kg)	4.80 (0.34)	4.53 (0.78)	4.74 (0.69)	4.54 (0.54)	4.52 (0.79)	4.13 (0.75)	0.461	0.205	0.974
Lamb birth weight (% of ewe body weight)	6.56 (0.58)	6.25 (0.56)	5.91 (0.98)	5.84 (0.88)	5.40 (0.85)	5.23 (1.30)	0.088	0.524	0.760
Lamb lung weight (g)	162 (15)	153 (19)	165 (23)	160 (27)	148 (36)	138 (30)	0.250	0.480	0.946
Lung weight (% of lamb weight)	3.3 (0.5)	3.4 (0.5)	3.5 (0.3)	3.6 (0.3)	3.3 (0.5)	3.4 (0.5)	0.326	0.711	0.479
Lamb brain weight (g)	56 (2)	56 (4)	54 (15)	61 (5)	58 (5)	58 (4)	0.666	0.320	0.314
Lamb brain weight (% of lamb weight)	0.12 (0.01)	0.12 (0.01)	0.13 (0.03)	0.14 (0.02)	0.13 (0.03)	0.15 (0.03)	0.085	0.254	0.922

*Note*: Data are means (SD) unless otherwise indicated. N/A, not analysed. One control twin died before delivery and was not included. ^a^Lambs were delivered by Caesarean section at 140 days of gestation (term = 150 days). ^b^Litter size is indicated: S, singleton; T, twin. ^c^Statistical significance (*P* < 0.05) is shown in bold. Although there was an overall effect of treatment, ewe body weight at delivery did not differ between any pairs of treatments.

### 3.2 Ventilation parameters

**FIGURE 2:**
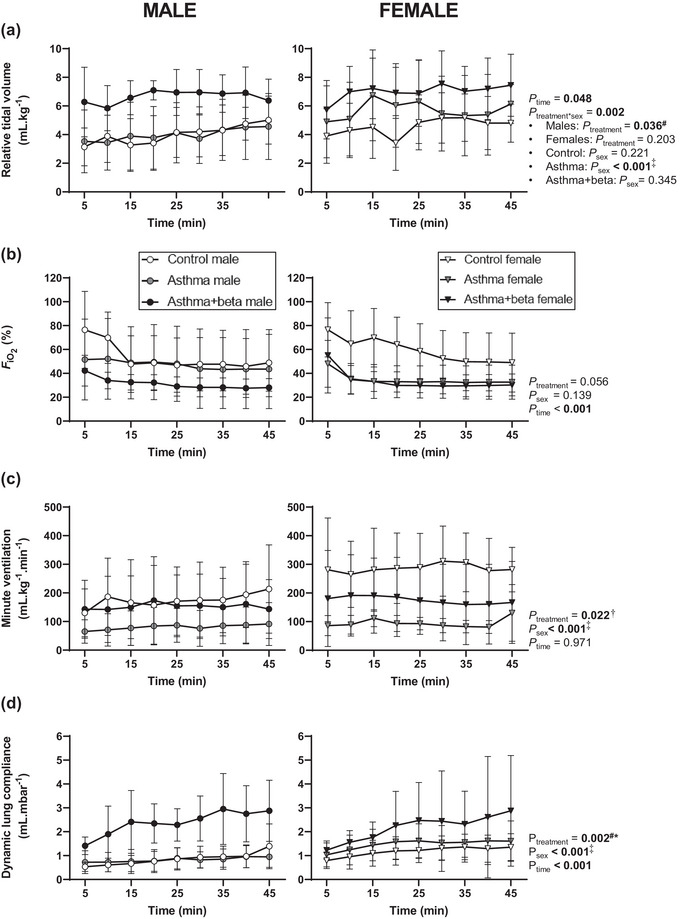
Ventilator parameters during the neonatal lung function study. Data are ventilator settings and output throughout the 45‐min ventilation period: relative tidal volume (a), fraction of inspired oxygen (b, $F_{iO_{2}}$), minute ventilation (c), and dynamic lung compliance (d). Data are from lambs born to control ewes (control, open symbols, male *n* = 8, female *n* = 7) asthmatic ewes (asthma, grey symbols, male *n* = 7, female *n* = 5) and lambs born to asthmatic ewes treated with antenatal betamethasone (asthma+beta, black symbols, male *n* = 4, female *n* = 7). Data are separated by sex: male lambs (circles, left panels) and female lambs (triangles, right panels). Data were analysed by mixed models, with time as a within‐animal factor, and are presented as means (SD). Statistically significant effects (*P* < 0.05) are shown in bold and interactions are reported when significant. Bonferroni correction was used to determine differences between groups where a treatment effect was significant. ^#^Controls < asthma+beta. ^†^Asthma ≠ control. *Asthma < asthma+beta. ^‡^Male < females.

The effects of treatment on tidal volume relative to lamb weight (Figure 2a) differed with lamb sex (interaction, *P* = 0.002), and relative tidal volume increased over time (*P *= 0.048). Treatment affected relative tidal volume in male (*P *= 0.036) but not female lambs (*P* = 0.203). In males, relative tidal volume was lower in control than asthma+beta lambs (*P* = 0.044), and not different in asthma and either asthma+beta (*P* = 0.103) or control lambs (*P *= 1.000). Relative tidal volume did not differ between sexes within the control (*P* = 0.221) or asthma+beta groups (*P* = 0.345) but was higher in females than males within asthma lambs (*P* < 0.001). The fraction of inspired oxygen required during ventilation ($F_{iO_{2}}$, Figure 2b) did not differ between treatments (*P* = 0.056; Figure 2b) or sexes (*P* = 0.151) and decreased over time (*P* < 0.001). Minute ventilation (Figure 2c) differed with treatment (*P *= 0.022) and sex (males < females; *P *< 0.001) and did not change over time (*P *= 0.971). Minute ventilation was lower in asthma than control lambs (*P* = 0.019) but did not differ between asthma+beta lambs and either control (*P* = 0.353) or asthma (*P* = 0.670) lambs. Dynamic lung compliance (Figure 2d) differed with treatment (*P *= 0.002) and sex (males < females; *P *< 0.001) and increased over time (*P *< 0.001). Compliance was higher in asthma+beta lambs than in either control (*P* = 0.003) or asthma (*P* = 0.008) lambs with no difference between lambs from asthma and control groups (*P* = 1.000).

### 3.3 Physiological parameters

**FIGURE 3:**
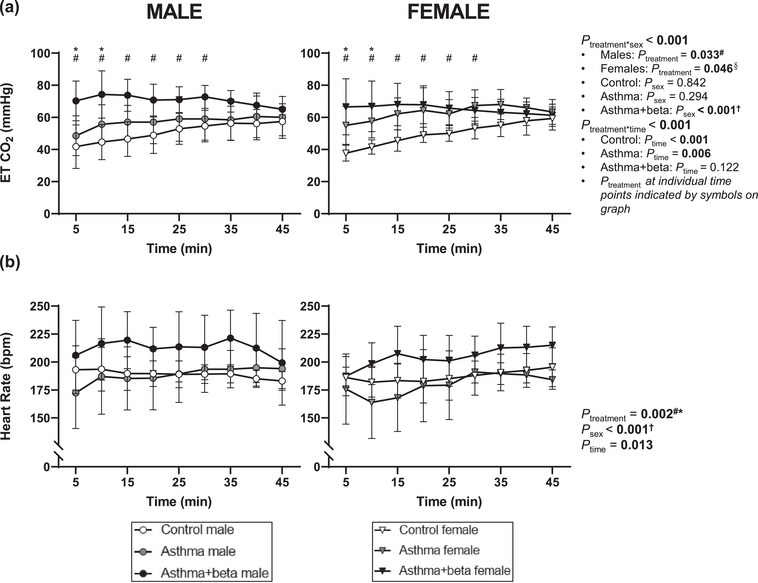
Physiological parameters in lambs during the neonatal lung function study. Data are from lambs born to control ewes (control, open symbols, male *n* = 8, female *n* = 7) asthmatic ewes (asthma, grey symbols, male *n* = 7, female *n* = 5) and lambs born to asthmatic ewes treated with antenatal betamethasone (asthma+beta, black symbols, male *n* = 4, female *n* = 7). Data show end‐tidal carbon dioxide (a) and heart rate (b). Data are separated by sex: male lambs (circles, left panel) and female lambs (triangles, right panel). Data are means (SD). Statistical significance (*P* < 0.05) is shown in bold, and interactions are reported when significant. Bonferroni correction was used to determine differences between groups where an overall treatment effect was significant. ^#^Controls ≠ asthma+beta. *Asthma < asthma+beta. ^†^Males > females. ^§^No significant differences after Bonferroni post‐hoc tests.

The effects of treatment on $ET_{CO_{2}}$ (Figure 3a) differed with lamb sex (interaction, *P* < 0.001) and time (interaction, *P* < 0.001). There was an effect of treatment on $ET_{CO_{2}}$ within males (*P *= 0.033) and females (*P *= 0.046). In males, asthma+beta had higher $ET_{CO_{2}}$ than control lambs (*P* = 0.031), but $ET_{CO_{2}}$ of asthma lambs did not differ from asthma+beta (*P* = 0.392) or controls (*P* = 0.597). In females, $ET_{CO_{2}}$ did not differ between control and asthma+beta (*P* = 0.114), control and asthma (*P* = 0.071), or asthma and asthma+beta groups (*P* = 1.000). $ET_{CO_{2}}$ did not differ between sexes in control (*P* = 0.842) or asthma lambs (*P* = 0.294) but was higher in males than females within asthma+beta lambs (*P* < 0.001). $ET_{CO_{2}}$ increased over time in control (*P* < 0.001) and asthma lambs (*P* = 0.006) but did not change with time in asthma+beta lambs (*P* = 0.122). Asthma+beta lambs had higher $ET_{CO_{2}}$ than control lambs from 5 until 30 min of the ventilation protocol, and higher $ET_{CO_{2}}$ than asthma lambs at 5 and 10 min (all *P* < 0.05); $ET_{CO_{2}}$ did not differ between control and asthma lambs at any time point. Heart rate (Figure 3b) differed between treatments (*P *= 0.002) and sexes (male > female, *P *< 0.001), and increased over time (*P *= 0.013). Heart rate was higher in asthma+beta lambs than in control (*P* = 0.007) or asthma lambs (*P* = 0.006) but was not different in asthma and control lambs (*P *= 1.000). Arterial partial pressures of oxygen and carbon dioxide are detailed with other blood biochemistry measures in the Supporting information (Figure S1).

**FIGURE 4:**
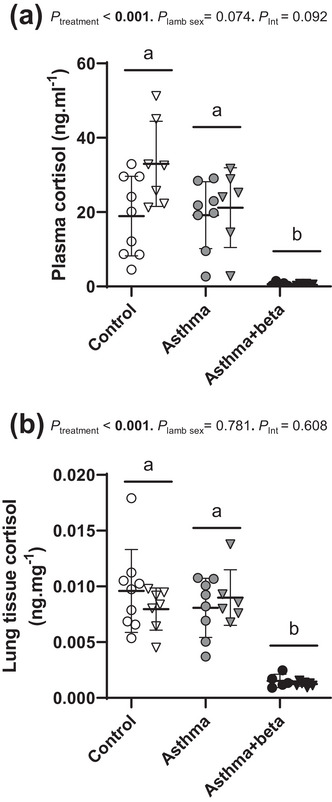
Plasma and lung tissue cortisol concentrations. Data show cortisol concentrations in plasma (a) and lung tissue (b). Data are separated by lamb sex into males (circles) and females (triangles). Data are from control lambs (open symbols, male *n* = 8–9, female *n* = 5–7), lambs born to asthmatic ewes (asthma, grey symbols, male *n* = 8, female *n* = 6), and lambs born to asthmatic ewes treated with antenatal betamethasone (asthma+beta, black symbols, male *n* = 4–5, female *n* = 6–7). Each symbol indicates data from one lamb, with whisker plots showing the mean ± SD for each group. Statistical significance (*P* < 0.05) is shown in bold. Bonferroni correction was used to determine differences between groups where an overall treatment effect was significant, indicated by different letters. Letters indicate differences between groups (a, b, *P* < 0.05). *P*_int_ = treatment × sex interaction analysis.

Cortisol concentrations in plasma (Figure 4a) differed with treatment (*P* < 0.001). Plasma cortisol concentrations were lower in asthma+beta lambs compared to control (*P* < 0.001) or asthma lambs (*P* < 0.001), and did not differ between lambs from asthma ewes and control ewes (*P* = 0.308). Cortisol concentrations in lung tissue collected at the end of the study (Figure 4b) differed with treatment (*P* < 0.001). Lung tissue cortisol concentrations were lower in asthma+beta lambs compared to control (*P* < 0.001) or asthma lambs (*P* < 0.001), and did not differ between lambs from asthma ewes and control ewes (*P = *1.000, respectively). Neither did cortisol concentrations differ between male and female lambs (*P* = 0.074 and *P *= 0.781, respectively, Figure 4). Antenatal betamethasone treatment similarly suppressed plasma cortisone, 11‐deoxycortisol and corticosterone concentrations and suppressed lung tissue corticosterone concentrations (Supporting information, Figure S2).

### 3.4 Lung structure and surfactant protein mRNA expression

**FIGURE 5:**
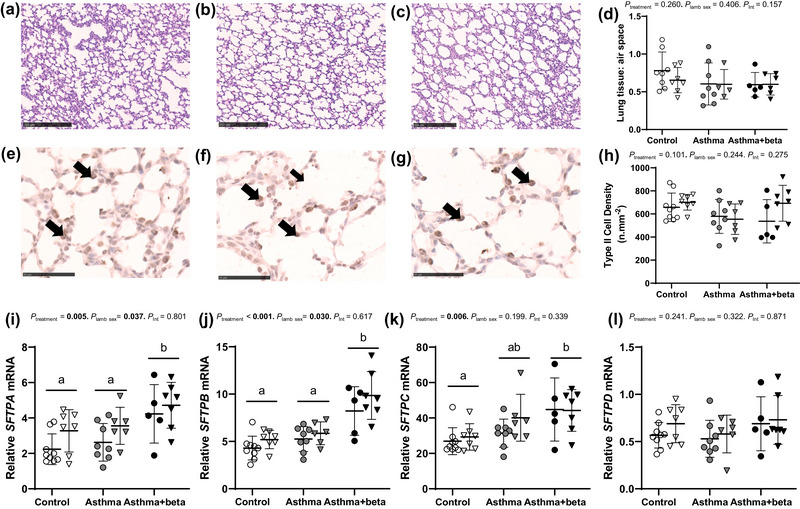
Lamb lung structural analyses and surfactant protein mRNA expression. Representative haematoxylin and eosin stained lamb lung sections (a–c, ×10 magnification, scale bars 250 µm, Hamamatsu NanoZoomer 2.0‐HT) and lung sections immunolabelled for surfactant protein B (e–g, ×60 magnification, scale bars 50 µm, Hamamatsu NanoZoomer 2.0‐HT) from control lambs (a, d, e, h, open symbols, male *n* = 8, female *n* = 7), lambs born to asthmatic ewes (b, d, f, h, asthma, grey symbols, male *n* = 8, female *n* = 4), and lambs born to asthmatic ewes treated with antenatal betamethasone (c, d, g, h, asthma+beta, black symbols, male *n* = 5, female *n* = 6). Data (d, h–l) are separated by sex: male lambs (circles) and female lambs (triangles). Four lambs were excluded from tissue‐to‐airspace analyses due to poor fixation of the lung. Type II cell density data points are means of counts from two tissue samples taken from inflation‐fixed left lamb lungs. Cells were only classified as type II cells (arrows) if they met all three criteria: SP‐B positive (brown cells), with a rounded morphology that bulged into alveolar space. SP‐B‐positive cells that did not meet colour, morphological and location criteria were not counted as type II cells. mRNA expression of *SFTPA* (i), *SFTPB* (j), *SFTPC* (k), and *SFTPD* (l) are relative to three stable housekeeping genes (*ACTB*, *HPRT1* and *TBP*). Data points (d, h–l) are means of data from two lung regions for each animal. Data are individual animals (symbols) with error bars showing means (SD). Statistical significance (*P* < 0.05) is shown in bold. Bonferroni correction was used to determine differences between groups (*P* < 0.05), indicated by different letters. *P*_int_ = treatment × sex interaction analysis. *ACTB*, beta‐actin; HRPT1, Hypoxanthine phosphoribosyltransferase; TBP, TATA box‐binding protein.

Tissue‐to‐airspace ratio (Figure 5d) was not affected by treatment (*P* = 0.260) or sex (*P* = 0.406). The density of type II cells (Figure 5h) was similarly unaffected by treatment (*P* = 0.101) or sex (*P* = 0.244). Expression of *SFTPA* (Figure 5i) and *SFTPB* (Figure 5j) differed with treatment (*P* = 0.005 and *P* < 0.001, respectively) and sex (females > males; *P* = 0.037 and *P* = 0.030, respectively). Expression of *SFTPA* and *SFTPB* was higher in asthma+beta than in control (*P* = 0.004 and *P* < 0.001 respectively) or asthma lambs (*P* = 0.048 and *P* < 0.001, respectively) and did not differ between asthma and control lambs (*P* = 1.000 and *P* = 0.895, respectively). Expression of *SFTPC* (Figure 5k) differed with treatment (*P* = 0.006) but not sex (*P* = 0.199). Expression of *SFTPC* was higher in asthma+beta than in control lambs (*P* = 0.005), and similar in asthma and control lambs (*P* = 0.381) or asthma+beta lambs (*P* = 0.177). Expression of *SFTPD* (Figure 5l) was unaffected by treatment (*P* = 0.241) or sex (*P* = 0.322). Gene expression of *HIF3A* and *SCNN1B* did not differ with treatment or sex, whilst gene expression of *KDR* differed with treatment but not sex (Supporting information, Figure S3).

## 4 DISCUSSION

The results of this study support our hypothesis that antenatal betamethasone may improve term neonatal lung function in the offspring of asthmatic mothers, in the context of a mild maternal phenotype. Betamethasone treatment increased dynamic lung compliance and *SFTPA* and *SFTPB* gene expression in lambs of asthmatic ewes compared to lambs from both control and untreated asthma ewes. Female lambs had higher dynamic lung compliance and expression of *SFTPA* and *SFTPB* than males. While maternal asthma did not impair neonatal lung function or surfactant gene expression in this study, our findings demonstrate that, even near term, antenatal betamethasone improves neonatal lung function, particularly in male offspring.

The lack of impact of maternal asthma on lamb surfactant gene expression differs from our previous study employing this model (Clifton et al., 2016), which may reflect a milder maternal asthma phenotype in the present cohort. In the present study, lung function and BAL eosinophil count in ewes, and lung compliance and type II cell density in near‐term lambs did not differ between control and asthma groups. Nevertheless, in the larger cohort of animals that included non‐pregnant ewes, dynamic lung function decreased more in asthmatic than control ewes between mating and ∼132 dG, indicating mild maternal asthma. The milder phenotype in these ewes differs from previous cohorts of sheep in which the same protocol was used to induce asthma, including in the context of pregnancy (Bischof et al., 2003; Clifton et al., 2016; Wooldridge et al., 2019). Neonatal outcomes, including fetal growth (Stevens et al., 2023), rates of preterm birth, nursery admission and congenital malformations, are improved by better control and less severe asthma in humans (Abdullah et al., 2020; Yland et al., 2020), and thus the milder asthma phenotype in the present study is likely to have less impact on neonates. Despite this, relative lamb birth weight was lower in progeny of asthmatic ewes compared to those from control ewes irrespective of betamethasone treatment, by a similar magnitude (−12.5%) as in our previous study in which ewes developed a more severe asthma phenotype (Clifton et al., 2016). Although ewes in both the current and previous studies were all Merinos, the South Australian strain from the present study was ∼2‐fold heavier than the Victorian strain in the original study, resulting in lower relative lung exposure to HDM, as the same dose was used (Clifton et al., 2016). The different ewe phenotypes may also reflect differences in immune function between animal cohorts, possibly reflecting prior or current pathogen and environmental exposures, or HDM batch differences. Given that the impacts of maternal asthma depend on the severity, the benefits of antenatal betamethasone are likely to be greater in the context of poorly controlled or severe maternal asthma, and decisions to treat should consider the maternal phenotype.

In lambs delivered near‐term, betamethasone treatment increased dynamic lung compliance and elevated *SFTPA* and *SFTPB* expression relative to saline‐treated asthma lambs and increased dynamic lung compliance and elevated *SFTPA*, *SFTPB* and *SFTPC* gene expression relative to controls. The hydrophobic *SFTPB* and *SFTPC* are the two surfactant proteins primarily responsible for reducing surface tension (Haagsman & Diemel, 2001), and increases in their expression likely contribute to better dynamic lung compliance in the betamethasone‐treated group. This upregulation of surfactant protein expression by antenatal betamethasone is similar to effects reported at earlier gestational ages. Antenatal betamethasone induced elevated *SFTPA* and *SFTPD* gene expression in lambs born at 96 dG (Visconti et al., 2018), while the gene expression of all four surfactant proteins (A–D) was increased at ∼124 dG in response to betamethasone treatment 24 and 48 h earlier (Schmidt et al., 2017). Betamethasone did not alter type II epithelial cell density in this cohort, consistent with findings in 125 dG sheep (Visconti et al., 2018). However, other responses to betamethasone differ between our study and previous reports, which may be due to the late gestational age of our lambs (140 dG; term ∼150 dG). The lack of betamethasone‐induced changes in lamb lung structure in the present study contrasts with reported increases in the proportion of airspace and decreases in the proportion of tissue at 124 dG (Polglase et al., 2007). The endogenous plasma cortisol surge commences at ∼134–137 dG in sheep (Orgeig et al., 2010; Phillips et al., 1996), which may have induced structural lung maturation before our antenatal treatments. Also contrasting with results of other studies, and potentially reflecting differences in gestational age, time from exposure or species, *HIF3A*, *KDR* and *SCNN1B* expression was not elevated in betamethasone‐exposed lambs compared to unexposed lambs in the present study, although betamethasone upregulates these genes in near‐term fetal rat lung fibroblasts 6 h after exposure and 94 dG sheep fetuses 48 h after exposure (Seow et al., 2019; Visconti et al., 2018).

A striking observation in the present study was the lower abundance of endogenous glucocorticoids in plasma and lung tissue in betamethasone‐exposed lambs, likely reflecting negative feedback of glucocorticoid receptor activation on endogenous cortisol production. Total circulating glucocorticoid activity, assessed first by radioreceptor assays and later by systems using cells transfected with reporter genes downstream of the human glucocorticoid receptor gene, is increased within 12 h and remains elevated for 1–5 days following maternal antenatal betamethasone treatment (Ballard et al., 1980; Kajantie et al., 2004). This increase reflects the activity of the exogenous steroid since cortisol and dehydroepiandrosterone concentrations in cord blood are suppressed within 1–6 h of maternal betamethasone injection to concentrations less than half those of infants that were untreated or last exposed more than 7 days previously (Ballard et al., 1980, 1975; Kajantie et al., 2004; Parker et al., 1996; Süvari et al., 2021). Circulating cortisol remains suppressed for up to 7 days after antenatal betamethasone (Ballard et al., 1980; Kajantie et al., 2004; Parker et al., 1996). Fewer data are available on the effects of antenatal dexamethasone on endogenous steroid production, although serum cortisol concentrations measured within 12 h of birth were ∼30% lower in neonates whose mothers had been treated with antenatal dexamethasone within 7 days of delivery (Karlsson et al., 2000). More detailed pharmacokinetic studies have recently been reported in women of reproductive age, whose plasma cortisol concentrations were suppressed until 60 h after a single 6 mg/mL intramuscular dose of betamethasone (Jobe et al., 2020). This suggests that exposure to antenatal betamethasone suppresses endogenous cortisol secretion for similar durations in the fetus, where concentrations can only be measured at delivery, as in adult women. Using liquid chromatography–tandem mass spectrometry methodology and avoiding potential issues of steroid cross‐reactivity in other assays, we have demonstrated even more profound suppression of circulating and lung cortisol concentrations in preterm lambs at 24 h after the second dose of antenatal betamethasone. Furthermore, we have extended these findings to also demonstrate that a single course of maternal antenatal betamethasone also suppresses concentrations of cortisol in fetal lung tissue, and reduces fetal circulating concentrations of additional endogenous steroids.

In the present study, male lambs had poorer respiratory outcomes and evidence of lung immaturity relative to females, with lower actual tidal volume, dynamic lung compliance and *SFTPA* and *SFTPB* gene expression, independent of treatment. If also present in humans, these sex differences may contribute to the greater risk of neonatal respiratory morbidity and poorer childhood lung function in preterm‐born males compared to preterm‐born females (Laube & Thome, 2022). Endocrine differences may underlie sexual dimorphism in normal lung maturation, with evidence in piglets that oestrogen and progesterone promote alveolar formation (Trotter et al., 2006), and studies in rabbits suggesting that androgens may inhibit fetal lung production of surfactants (Nielsen et al., 1982).

Our data also support the hypothesis that responses to ACS differ between sexes. Relative tidal volume and $ET_{CO_{2}}$ were higher in asthma+beta compared to control males but did not differ between asthma+beta and control females. Conversely, arterial pH and acid base excess were higher in asthma+beta compared to control females but did not differ between asthma+beta and control males. In the present study, the effects of betamethasone on lung compliance were similar in male and female lambs delivered at 140 dG, in contrast to a study of 128 dG preterm lambs, where benefits of betamethasone on lung compliance were greater in females than males (Willet et al., 1997). Overall, the smaller benefits in females in the present study likely reflect greater maturation of the female lung in the near‐term sheep, as discussed above, lessening the opportunity for further responses to exogenous corticosteroids. Clinically, evidence for sex differences in response to ACS is mixed, with improved survival and reduced rates of intubation and incidence of bronchopulmonary dysplasia in preterm (<29 weeks gestation) males but not females in one study (Ramos‐Navarro et al., 2022), although ACS reduced the risk of RDS in preterm (28–33 weeks) females but not males in another study (Collaborative Group on Antenatal Steroid Therapy, 1981). Additional comparisons between sexes across gestational ages are needed to determine sex differences in response in humans.

Strengths of the study include using sheep as a preclinical model of pregnancy complications and for testing antenatal interventions, since the maturity of the sheep lung at birth is more comparable to the lungs of humans than rats or mice (Lock et al., 2013; Morrison et al., 2018). Maternal asthma was induced using a well‐established protocol (Bischof et al., 2003; Clifton et al., 2016; Wooldridge et al., 2019) and key characteristics of the asthma phenotype were measured (Robinson, Gatford, Bailey, et al., 2024), although the maternal asthma phenotype was milder than previously reported (Clifton et al., 2016). Limitations of this study include a potential lack of statistical power to detect differences, since not every treatment group contained seven lambs per sex, which we calculated would be required to detect a 20% change (at 80% power) in surfactant gene expression and alveolar cell densities based on our previous studies (Clifton et al., 2016; Wooldridge et al., 2019). Nevertheless, effects of antenatal betamethasone were clearly evident. An additional limitation of the study includes the unexpectedly high proportion of twin pregnancies, which introduced an additional source of fetal growth restriction (Muhlhausler et al., 2011) and thus may have reduced the ability to detect structural and functional lung differences, in contrast to our previous singleton‐only study (Clifton et al., 2016). Additionally, some baseline lung ventilation parameters in the controls differed from those previously reported in studies of ventilated lambs of a similar gestational age (Schmölzer et al., 2022; Yamaoka et al., 2022), with greater acidosis, hypercapnia and oxygen demand. We did not include a group of control ewes treated antenatally with betamethasone in the present study since we were evaluating an intervention to improve neonatal respiratory outcomes in the setting of maternal asthma and antenatal betamethasone is not currently clinically recommended for use in women expected to deliver at term (Reddy et al., 2021).

An improvement in neonatal dynamic lung compliance, induced by betamethasone treatment of asthmatic mothers, may reduce the risk of lung disease at birth, particularly in males. We suggest that the long‐term consequences of betamethasone should also be studied to determine if any benefit for newborn lung function is sustained given that adverse respiratory outcomes associated with maternal asthma are also seen in childhood (Berry et al., 2016; Lim et al., 2010). At present, ACS is clinically advised only before 37 weeks of gestation, where they benefit lung maturation and short‐term morbidity (Malaeb & Stonestreet, 2014; Reddy et al., 2021). Before ACS use is extended to term births in mothers with asthma, we also suggest that long‐term neurological outcomes should be investigated given concerns regarding the adverse effects of ACS treatment on childhood neurodevelopment (Ninan et al., 2022; Vidaeff et al., 2023). Our data also provide evidence that mild maternal asthma does not impair neonatal lung function. This is consistent with clinical evidence that good control of maternal asthma during pregnancy improves offspring respiratory health (Collier et al., 2013; Liu et al., 2018; Martel et al., 2009; Morten et al., 2018). Together, this suggests that improving maternal asthma control during pregnancy should be a priority, while antenatal betamethasone may be an effective rescue strategy to improve neonatal respiratory health where maternal asthma is not well‐controlled.

## AUTHOR CONTRIBUTIONS

Conception or design of the work: Kathryn L. Gatford, Janna L. Morrison, Michael J. Stark, Andrew Tai, Beverly S. Muhlhausler, Robert J. Bischof, Vicki L. Clifton, Tim J. M. Moss and Megan J. Wallace. Experiments were performed by: Joshua L. Robinson, Andrea J. Roff, Sarah J. Hammond, Jack R. T. Darby, Stacey L. Holman, Ashley S. Meakin, Catherine G. Dimasi, Sarah M. Jesse, Kathryn L. Gatford, Michael J. Stark and Janna L. Morrison. Acquisition, analysis, or interpretation of data for the work: Joshua L. Robinson, Sarah M. Jesse, Sarah J. Hammond, Jack R. T. Darby, Stacey L. Holman, Ashley S. Meakin, Michael D. Wiese, Kathryn L. Gatford, Janna L. Morrison, Megan J. Wallace, Andrew N. Davies and Michael J. Stark. Drafting the work or revising it critically for important intellectual content: Joshua L. Robinson, Kathryn L. Gatford, Janna L. Morrison, Megan J. Wallace and Michael J. Stark. All authors have read and approved the final version of this manuscript and agree to be accountable for all aspects of the work in ensuring that questions related to the accuracy or integrity of any part of the work are appropriately investigated and resolved. All persons designated as authors qualify for authorship, and all those who qualify for authorship are listed.

## CONFLICT OF INTEREST

The authors declare no conflicts of interest.

## Supporting information



Figure S1. Blood chemistry in lambs during the neonatal lung function study.
Figure S2. Plasma and lung tissue glucocorticoid concentrations.
Figure S3. HIF3A, KDR and SCNN1B gene expression.

Supporting information

Supporting information

Supporting information

## Data Availability

This article has an online data supplement containing further details on methods including induction of the maternal asthma phenotype, ewe lung function measures, histological analyses, real‐time PCR and glucocorticoid analyses. The online data supplement also includes additional results on the blood biochemistry of lambs during ventilation, plasma and lung tissue glucocorticoid concentrations, and *HIF3A*, *KDR* and *SCNN1B* gene expression in lungs. The data generated and analysed during this study have been uploaded as supplementary files or is available from the corresponding author on reasonable request.
