# Supplementary material for: Betamethasone improved near‐term neonatal lamb lung maturation in experimental maternal asthma
Source: Exp Physiol. 2024 Oct 22;109(11):1967–79. doi: 10.1113/EP091997 (PMC11522833; doi:10.1113/EP091997)
Supplement: Supplementary file 1 — Figure S1. Blood chemistry in lambs during the neonatal lung function study. Figure S2. Plasma and lung tissue glucocorticoid concentrations. Figure S3. HIF3A, KDR and SCNN1B gene expression. [file EPH-109-1967-s004.docx]

**Betamethasone improved near-term neonatal lamb lung maturation in experimental maternal asthma**

Joshua L. Robinson, Andrea J. Roff, Sarah J. Hammond, Jack R. T. Darby, Ashley S. Meakin, Stacey L. Holman, Andrew Tai, Tim J. M. Moss, Catherine G. Dimasi, Sarah M.

Jesse, Michael D. Wiese, Andrew N. Davies, Beverly S. Muhlhausler, Robert J. Bischof, Megan J. Wallace, Vicki L. Clifton, Janna L. Morrison, Michael J. Stark, and Kathryn L. Gatford

ONLINE DATA SUPPLEMENT

**Supplementary methods**

*Animals and induction of maternal asthma phenotype*

Animals were housed in paddocks, grazing natural pastures with supplemental hay as needed, throughout the experiment. Merino ewes (3 years old, primiparous, n = 65; Fig 1) were randomised using a random number generator into control (n = 15; Fig 1) and house dust mite (HDM) sensitisation (allergic, n = 50; Fig 1) groups. Ewes were sensitised by five subcutaneous injections of HDM extract (*Dermatophagoides pteronyssinus*, 1 mL injection, 50 μg.mL^-1^ in alum) at two-week intervals (Bischof *et al.*, 2003). Blood samples were taken before commencing sensitisations and seven days after the final sensitisation injection, and HDM-specific IgE levels were measured in plasma by enzyme-linked immunosorbent assay (Bischof *et al.*, 2008; Wooldridge *et al.*, 2014). HDM-exposed sheep with a ≥1.5-fold increase in HDM-specific IgE were considered sensitised (n = 26; Fig 1). Non-sensitised sheep (n = 24) were either returned to the commercial flock (n = 19; Fig 1) or transferred to the control group (n = 5; Fig 1).

To induce the asthmatic phenotype, fortnightly airway challenges with saline (control ewes) or HDM (sensitised ewes) were given until the end of the experiment (Bischof *et al.*, 2003) over 10 weeks of challenges prior to mating and 20 weeks of challenges after mating. Each challenge was delivered into the lung through a catheter guided by a fibre-optic bronchoscope (Olympus GIF-N30, VIC, Australia) with 5 mL of saline (control group) or 5 mL of HDM (200 μg.mL^-1^, allergic group) instilled into each of the left and right main bronchi (Bischof *et al.*, 2003).

*Ewe lung function measures*

Ewe lung function was measured in late pregnancy (~132 days of gestation, dG). An endotracheal (ET) tube, an oesophageal catheter and a tracheal catheter were inserted via the nostrils. Ewes were not sedated as per previous protocols (Clifton *et al.*, 2016) given the ewes were calm in a custom-made harness; Lignocaine gel (Xylocaine 2% Jelly, Aspen Pty Ltd, NSW, Australia) was applied to both nostrils of the sheep to minimise discomfort. The catheters were attached to differential pressure transducers to determine transpulmonary pressures and the ET tube was connected to a pneumotachometer and spirometer to measure respiratory flow, and all measures were recorded in LabChart 7 (ADInstruments Pty Ltd, Bella Vista, NSW, Australia). Dynamic compliance was derived from breath-by-breath analysis of intra- and extra-thoracic pressures during regular breathing periods in resting sheep (Koumoundouros *et al.*, 2006). Lung function for each ewe was determined in at least five epochs of 4-5 breaths collected during quiet breathing across a minimum of 10 minutes (Koumoundouros *et al.*, 2006).

Before and 48 h after lung function and challenge (~132 dG), bronchoalveolar lavage (BAL) was collected via a catheter placed in the biopsy port of the bronchoscope (20 mL saline infused; 1.9-14 mL lavage recovered). The recovered BAL fluid was centrifuged at 400 *g* for 7 minutes to separate BAL cells and supernatant. BAL cells were fixed and stained in Turk’s solution (Merck Millipore, Melbourne, VIC, Australia) and total cells were counted on a haemocytometer. BAL samples for differential cell counts were prepared on microscope slides, stained with Kwik-Diff (Thermo Electron Corp, Waltham, MA, USA) and light microscopy was used to determine the number of eosinophils, a marker of allergic asthma, relative to total numbers of leukocytes per mL of BAL (Liravi *et al.*, 2015).

*Delivery and ventilation*

At 140 ± 2 dG (term = 150 dG), fasted ewes were anaesthetised intravenously with ketamine (7 mL.kg^-1^) and diazepam (0.3 mL.kg^-1^), intubated, and anaesthesia of the ewe and fetus maintained with isoflurane (1.5-2.5% in air; Lyppards, SA, Australia). Lambs were intubated and one carotid artery and one jugular vein were catheterised, prior to delivery by Caesarean section. To assess lung function, lambs were mechanically ventilated (Babylog 8000 Plus, Dräger Medical) for 45 minutes using a volume guarantee strategy of 7 mL.kg^-1^, starting peak inflation pressure (PIP) 25 cmH_2_O, and positive end-expiratory pressure (PEEP) 5-6 cmH_2_O (Polglase *et al.*, 2015). Anaesthesia of lambs during the ventilation study was maintained by a continuous intravenous infusion of alfaxalone (5-15 mg.kg^-1^, Alfaxan-CD RTU; Jurox Pty. Ltd., Rutherford, New South Wales, Australia). Ventilator settings were adjusted throughout the protocol to target O_2_ saturation of 95-98% and end-tidal CO_2_ of 45-50 mmHg, measured using a mainstream carbon dioxide sensor. Ventilator settings and outputs, as well as core body temperature, heart rate, respiratory rate, and peripheral oxygen saturation (S_p_O_2_) from pulse oximetry, were recorded every five minutes. Arterial blood (0.2-0.5 mL) was sampled prior to delivery and then every five minutes during ventilation and blood gases were analysed with a RAPIDPOINT 500 machine (Siemens Healthineers, Erlangen, Germany) with temperature corrected to 39°C. Arterial and venous catheters were connected to LabChart 7 (ADInstruments, Bella Vista, NSW, Australia) for continuous blood pressure monitoring.

*Neonatal lung structure*

To assess lung tissue-to-airspace ratio, one cranial and one caudal lung section were stained with haematoxylin and eosin (McGillick *et al.*, 2016) and point counted, while blinded to treatment, using Visiopharm software (Hoersholm, Denmark). Briefly, 20 fields of view (FOV) with 100 points per FOV at 10x magnification were selected by random-systematic sampling across the entire tissue section and any FOV with ≥25% features other than air space or tissue was excluded (Westover *et al.*, 2012). For each included FOV, each point was labelled as tissue, airspace or other (defined as any point not alveolar airspace or alveolar walls, such as artefacts).

To determine the density of surfactant-producing type II alveolar epithelial cells, immunohistochemistry was performed using an anti-surfactant protein B (SP-B) antibody (Lock *et al.*, 2015). The primary antibody was a rabbit anti-mature SP-B antibody (1:500, Seven Hills Bioscience, # WRAB-48604). The secondary antibody was a biotin-labelled goat anti-rabbit antibody (1:100, IgG (H+L) Thermofisher, # 65-6140). Streptavidin-horseradish peroxidase conjugate (100 μg.mL^-1^, Invitrogen) and 3,3′-Diaminobenzidine (100 μg.mL^-1^, DAB, Invitrogen) chromagen enabled visualisation of SP-B-positive cells. Sections were counter-stained with Mayer’s Haematoxylin (Sigma-Aldrich). Negative controls used rabbit serum instead of primary antibody, and additional controls omitted the primary antibody. For each lung section, 50 FOVs per lung section were point-counted in Visiopharm at 60x magnification, selected by random systematic sampling and blinded to treatment. Type II alveolar epithelial cells met all three of the following criteria: rounded morphology, projection into alveolar space and stronger SP-B positive (SP-B+) DAB staining compared to other cells (Flecknoe *et al.*, 2000). The density of Type II cells per mm^2^ of lung tissue was calculated as previously described (Lock *et al.*, 2015).

*Real-time PCR*

All essential information regarding the qRT-PCR procedure is included as per the MIQE guidelines (Bustin *et al.*, 2009). Total RNA was extracted from frozen lung tissue for each fetus using QIAzol Lysis Reagent solution and QIAgen miRNeasy purification columns, as per manufacturer guidelines (Qiagen, Germany). Total RNA was quantified, and integrity was checked by spectrophotometric measurements at 260 and 280 nm in a NanoDrop Lite Spectrophotometer (Thermo Fisher Scientific). cDNA was synthesised using Superscript III First Strand Synthesis System (Invitrogen, USA) using 1 µg of total RNA, random hexamers, dNTP, DTT and Superscript III in a final volume of 20 µL, as per the manufacturer’s guidelines in an Applied Biosystems SimpliAmp Thermocycler (Thermo Fisher Scientific, Thebarton, South Australia). Controls containing no RNA transcript or no Superscript III were used to test for reagent contamination and genomic DNA contamination, respectively. The geNorm component of qbaseplus 2.0 software (Biogazelle, Belgium) was used to determine the most stable reference genes from a panel of candidate reference genes (Vandesompele *et al.*, 2002) and the minimum number of reference genes required to calculate a stable normalisation factor (Soo *et al.*, 2012; McGillick *et al.*, 2013; Lie *et al.*, 2014). For qRT-PCR data output normalisation, three stable reference genes: *BACTIN*, *HPRT*, and *TBP* were run in parallel with all target genes (Lock *et al.*, 2017). Primers were validated and optimised as previously described (Orgeig *et al.*, 2010; McGillick *et al.*, 2014; McGillick *et al.*, 2021). Relative expression of surfactant protein gene (*SFTPA*, *SFTPB, SFTPC, SFTPD*) targets (Orgeig *et al.*, 2010; McGillick *et al.*, 2013) were measured by qRT-PCR (Lock *et al.*, 2017) using KiCqStart SYBR Green qPCR ReadyMix (Sigma Aldrich, USA) in a final volume of 6 µL on an Applied Biosystems QuantStudio 7 Pro (Thermo Fisher Scientific, Thebarton, South Australia). Each qRT-PCR well contained 3 µL SYBR Green Master Mix (2X), 2 µL of forward and reverse primer mixed with H_2_O to obtain final primer concentrations and 1 µL of diluted cDNA. Each sample was run in triplicate for target and reference genes. The abundance of each transcript relative to the abundance of stable reference genes (Hellemans *et al.*, 2007) was calculated and expressed as mRNA mean normalised expression (MNE). No outliers were detected using the Grubbs method (GraphPad Prism 8, USA).

*Plasma and lung tissue glucocorticoid assay*

Plasma and lung tissue glucocorticoids in each lamb were determined by mass spectrophotometry as previously described (Dimasi *et al.*, 2023; Lock *et al.*, 2023). Plasma was from an arterial blood sample collected prior to delivery; lung tissue was homogenised and centrifuged prior to liquid-liquid extraction. A 100 μL aliquot of the supernatant from the tissue homogenate, or of plasma, was added to 300 μL acetonitrile, along with an internal standard (50 ng.mL^−1^ cortisol-9,11,12,12-d4; Toronto Research Chemicals, Toronto, Canada), before vortex and centrifugation. The supernatant was transferred to a new tube before adding 300 μL ethyl acetate, vortex, and recentrifugation and adding the resulting supernatant to the previously collected supernatant. Samples were dried using the GeneVac EZ-2 Evaporating System (GeneVac, UK), and then reconstituted in 50% methanol. Hormone concentrations were determined by liquid chromatography (LC; Shimadzu Nexera XR, Shimadzu, Japan) coupled to a SCIEX 6500 Triple-Quad system (MS/MS; SCIEX, US) and then injected into an ACQUITY UPLC BEH C18 Column 130Å, 1.7 μm, 2.1 mm × 100 mm (Waters Corp, USA). Mobile phases were 0.1% formic acid in water and 0.1% formic acid in acetonitrile. Flow rates and times, and detection of analytes were as previously described (McBride *et al.*, 2021; Dimasi *et al.*, 2023; Lock *et al.*, 2023). Hormone concentrations were calculated via integration with a standard curve that ranged from 0.05 ng.mL^−1^ to 100 ng.mL^−1^ (limit of quantitation = 0.01 ng.mL^−1^ for all hormones). When glucocorticoid data were below detectable limits, the value of the lowest limit of quantitation was used. Any missing glucocorticoid data reflect samples where duplicate analyses were too variable for integration into the standard curve. Tissue hormone concentrations were normalised to the tissue weight used for extraction.

**Supplementary results**

*Blood biochemistry*

The effects of treatment on arterial pH (Fig S1A) differed between sexes (interaction, *P* = 0.005) and with time (interaction, *P* = 0.001). Arterial pH differed between treatments in females (*P =* 0.007) but not in males (*P* = 0.275). In females, pH was higher in asthma+beta than control lambs (*P* = 0.007) but did not differ between asthma+beta and asthma (*P* = 0.991), or between control and asthma lambs (*P =* 0.058). Arterial pH was higher in males than females within control lambs (*P* < 0.001) but did not differ between sexes within asthma (*P* = 0.246) or asthma+beta groups (*P* = 0.938). pH did not change with time in control (*P* = 0.244) or asthma lambs (*P* = 0.783), whilst pH increased over time in asthma+beta lambs (*P* = 0.002). Arterial pH was higher in asthma+beta than control lambs from 20 to 45 minutes (all *P* < 0.050) but was not different at any time point between control and asthma lambs or asthma+beta and asthma lambs. The effects of treatment on actual base excess (ABE, Fig S1B) differed with sex (interaction, *P* = 0.002) and ABE increased over time (*P* < 0.001). ABE differed with treatment in females (*P* = 0.024), but not in males (*P* = 0.487). In females, ABE was higher in asthma+beta than control lambs (*P* = 0.035) but not different between control and asthma lambs (*P* = 0.069) or between asthma+beta and asthma lambs (*P* = 1.000). ABE was higher in males than females within control (*P* < 0.001), lower in males than females within asthma (*P* < 0.001), and not different between sexes within asthma+beta lambs (*P* = 0.595). Arterial lactate (Fig S1C) did not differ between treatments (*P* = 0.626), was higher in females than males (*P* = 0.002) and decreased over time (*P* < 0.001). Arterial oxygen saturation (Fig S1D) did not differ between treatments (*P* = 0.632) or sexes (*P* = 0.114) and increased from the fetal to postnatal period (*P* < 0.001). Similarly, the arterial partial pressure of oxygen (P_a_O_2_, Fig S1E) did not differ with treatment (*P* = 0.446) or sex (*P* = 0.095) and increased from the fetal to postnatal period (*P* < 0.001). Effects of treatment on arterial partial pressure of carbon dioxide (P_a_CO_2_, Fig S1F) differed with time (interaction, *P* = 0.016), whilst P_a_CO_2_ was unaffected by sex (*P* = 0.139). P_a_CO_2_ increased from the fetal period and throughout the postnatal period in control lambs (*P* = 0.002), decreased in the postnatal period over time in asthma+beta lambs (*P* = 0.043), and did not change with time in asthma lambs (*P* = 0.321). PaCO_2_ was lower in asthma+beta than control lambs at 25, 40, and 45 minutes, and did not differ between other treatment pairs or at other time points. Raw data for Figure S1 are available in the supporting information.

*Plasma and tissue glucocorticoid concentrations*

Plasma cortisone (Fig S2A) and 11-deoxycortisol (Fig S2B) concentrations differed with treatment (*P* < 0.001 and *P* = 0.024 respectively), but were unaffected by sex (*P* = 0.332, and *P* = 0.685 respectively). Plasma concentrations of cortisone did not differ between control and asthma lambs (P = *P =* 1.000) and were lower in asthma+betamethasone lambs compared to control (*P* < 0.001) or asthma lambs (*P* < 0.001). Plasma 11-deoxycortisol was lower in asthma+beta lambs compared to control (*P* = 0.023) but not asthma lambs (*P* = 0.155) and did not differ between control and asthma lambs (*P* = 1.000). The effect of treatment on plasma corticosterone concentration differed with sex (interaction: *P* = 0.035). Plasma corticosterone differed between sexes in the control group (*P* = 0.023, male < female) but not in asthma (*P* = 0.835) or asthma+beta lambs (*P* = 0.428). Plasma corticosterone concentration (Fig S2C) differed with treatment within both male (*P* = 0.018) and female lambs (*P* < 0.001). In males, plasma corticosterone was lower in asthma+beta than control (*P* = 0.020) but not asthma lambs (*P* = 0.052) and not between control and asthma (*P* = 1.000). In females, plasma corticosterone was lower in asthma+beta than control (*P* < 0.001) but not asthma lambs (*P* = 0.311) and was higher in controls than asthma (*P* = 0.033).

The effect of treatment on lung tissue cortisone (Fig S2D) differed with sex (interaction: *P* = 0.030). Lung tissue cortisone concentration differed between sexes in the control group (*P* = 0.007, male > female) but not in asthma (*P* = 0.173) or asthma+beta lambs (*P* = 0.785). Lung tissue cortisone differed with treatment in females (*P* = 0.006) but not males (*P* = 0.065). In females, lung tissue cortisone was lower in asthma+beta than asthma lambs (*P* = 0.004) but did not differ between asthma+beta and control lambs (*P* = 0.322), nor between control and asthma lambs (*P* = 0.140). Lung tissue 11-deoxyycortisol concentration (Fig S2E) differed with treatment (*P* = 0.049), but not within groups, and did not differ between sexes (*P* = 0.238). Nevertheless, lung tissue 11-deoxyycortisol concentration did not differ between asthma+beta compared to control (*P* = 0.051) or asthma (*P* = 0.219), or between control and asthma lambs (*P* = 1.000). Lung tissue corticosterone (Fig S2F) differed with treatment (*P* < 0.001) but not with sex (*P* = 0.308). Lung tissue corticosterone was lower in asthma+beta lambs compared to control (*P* < 0.001) and asthma lambs (*P* = 0.005) and did not differ between control and asthma lambs (*P* = 0.978).

*Further gene expression data*

*HIF3A* and *SCNN1B* gene expression (Fig S3A,C) did not differ with treatment (*P =* 0.116 and *P* = 0.455 respectively) or sex (*P =* 0.116 and *P* = 0.455 respectively). *KDR* gene expression differed with treatment (*P =* 0.014) but not sex (*P =* 0.756). *KDR* (Fig S3B) gene expression was lower in asthma+beta lambs compared to control (*P* = 0.012) but not asthma lambs (*P* = 0.305) and did not differ between control and asthma lambs (*P* = 0.382).

**References**

Bischof RJ, Snibson K, Shaw R & Meeusen EN. (2003). Induction of allergic inflammation in the lungs of sensitized sheep after local challenge with house dust mite. *Clin Exp Allergy* **33,** 367-375.

Bischof RJ, Snibson KJ, Van Der Velden J & Meeusen EN. (2008). Immune response to allergens in sheep sensitized to house dust mite. *J Inflamm (Lond)* **5,** 16.

Bustin SA, Benes V, Garson JA, Hellemans J, Huggett J, Kubista M, Mueller R, Nolan T, Pfaffl MW, Shipley GL, Vandesompele J & Wittwer CT. (2009). The MIQE guidelines: minimum information for publication of quantitative real-time PCR experiments. *Clin Chem* **55,** 611-622.

Clifton VL, Moss TJ, Wooldridge AL, Gatford KL, Liravi B, Kim D, Muhlhausler BS, Morrison JL, Davies A, De Matteo R, Wallace MJ & Bischof RJ. (2016). Development of an experimental model of maternal allergic asthma during pregnancy. *J Physiol* **594,** 1311-1325.

Dimasi CG, Darby JRT, Cho SKS, Saini BS, Holman SL, Meakin AS, Wiese MD, Macgowan CK, Seed M & Morrison JL. (2023). Reduced in utero substrate supply decreases mitochondrial abundance and alters the expression of metabolic signalling molecules in the fetal sheep heart. *J Physiol***,** Advance online publication. <https://doi.org/10.1113/JP285572>.

Flecknoe S, Harding R, Maritz G & Hooper SB. (2000). Increased lung expansion alters the proportions of type I and type II alveolar epithelial cells in fetal sheep. *Am J Physiol Lung Cell Mol Physiol* **278,** L1180-1185.

Hellemans J, Mortier G, De Paepe A, Speleman F & Vandesompele J. (2007). qBase relative quantification framework and software for management and automated analysis of real-time quantitative PCR data. *Genome Biol* **8,** R19.

Koumoundouros E, Bischof RJ, Meeusen EN, Mareels IM & Snibson KJ. (2006). Chronic airway disease: deteriorating pulmonary function in sheep associated with repeated challenges of house dust mite. *Exp Lung Res* **32,** 321-330.

Lie S, Morrison JL, Williams-Wyss O, Suter CM, Humphreys DT, Ozanne SE, Zhang S, MacLaughlin SM, Kleemann DO, Walker SK, Roberts CT & McMillen IC. (2014). Impact of embryo number and maternal undernutrition around the time of conception on insulin signaling and gluconeogenic factors and microRNAs in the liver of fetal sheep. *Am J Physiol Endocrinol Metab* **306,** E1013-1024.

Liravi B, Piedrafita D, Nguyen G & Bischof RJ. (2015). Dynamics of IL-4 and IL-13 expression in the airways of sheep following allergen challenge. *BMC Pulm Med* **15,** 101.

Lock MC, Botting KJ, Allison BJ, Niu Y, Ford SG, Murphy MP, Orgeig S, Giussani DA & Morrison JL. (2023). MitoQ as an antenatal antioxidant treatment improves markers of lung maturation in healthy and hypoxic pregnancy. *J Physiol* **601,** 3647-3665.

Lock MC, McGillick EV, Orgeig S, McMillen IC, Mühlhäusler BS, Zhang S & Morrison JL. (2017). Differential effects of late gestation maternal overnutrition on the regulation of surfactant maturation in fetal and postnatal life. *J Physiol* **595,** 6635-6652.

Lock MC, McGillick EV, Orgeig S, Zhang S, McMillen IC & Morrison JL. (2015). Mature surfactant protein-B expression by immunohistochemistry as a marker for surfactant system development in the fetal sheep lung. *J Histochem Cytochem* **63,** 866-878.

McBride GM, Meakin AS, Soo JY, Darby JRT, Varcoe TJ, Bradshaw EL, Lock MC, Holman SL, Saini BS, Macgowan CK, Seed M, Berry MJ, Wiese MD & Morrison JL. (2021). Intrauterine growth restriction alters the activity of drug metabolising enzymes in the maternal-placental-fetal unit. *Life Sci* **285,** 120016.

McGillick EV, Morrison JL, McMillen IC & Orgeig S. (2014). Intrafetal glucose infusion alters glucocorticoid signaling and reduces surfactant protein mRNA expression in the lung of the late-gestation sheep fetus. *Am J Physiol Regul Integr Comp Physiol* **307,** R538-545.

McGillick EV, Orgeig S, Allison BJ, Brain KL, Niu Y, Itani N, Skeffington KL, Kane AD, Herrera EA, Morrison JL & Giussani DA. (2021). Molecular regulation of lung maturation in near-term fetal sheep by maternal daily vitamin C treatment in late gestation. *Pediatr Res* **91,** 828-838.

McGillick EV, Orgeig S, McMillen IC & Morrison JL. (2013). The fetal sheep lung does not respond to cortisol infusion during the late canalicular phase of development. *Physiol Rep* **1,** e00130.

McGillick EV, Orgeig S & Morrison JL. (2016). Structural and molecular regulation of lung maturation by intratracheal vascular endothelial growth factor administration in the normally grown and placentally restricted fetus. *J Physiol* **594,** 1399-1420.

Orgeig S, Crittenden TA, Marchant C, McMillen IC & Morrison JL. (2010). Intrauterine growth restriction delays surfactant protein maturation in the sheep fetus. *Am J Physiol Lung Cell Mol Physiol* **298,** L575-583.

Polglase GR, Dawson JA, Kluckow M, Gill AW, Davis PG, Te Pas AB, Crossley KJ, McDougall A, Wallace EM & Hooper SB. (2015). Ventilation onset prior to umbilical cord clamping (physiological-based cord clamping) improves systemic and cerebral oxygenation in preterm lambs. *PLoS One* **10,** e0117504.

Soo PS, Hiscock J, Botting KJ, Roberts CT, Davey AK & Morrison JL. (2012). Maternal undernutrition reduces P-glycoprotein in guinea pig placenta and developing brain in late gestation. *Reprod Toxicol* **33,** 374-381.

Vandesompele J, De Preter K, Pattyn F, Poppe B, Van Roy N, De Paepe A & Speleman F. (2002). Accurate normalization of real-time quantitative RT-PCR data by geometric averaging of multiple internal control genes. *Genome Biol* **3,** Research0034.

Westover AJ, Hooper SB, Wallace MJ & Moss TJ. (2012). Prostaglandins mediate the fetal pulmonary response to intrauterine inflammation. *Am J Physiol Lung Cell Mol Physiol* **302,** L664-678.

Wooldridge AL, Bischof RJ, Meeusen EN, Liu H, Heinemann GK, Hunter DS, Giles LC, Kind KL, Owens JA, Clifton VL & Gatford KL. (2014). Placental restriction of fetal growth reduces cutaneous responses to antigen after sensitization in sheep. *Am J Physiol Regul Integr Comp Physiol* **306,** R441-446.

**
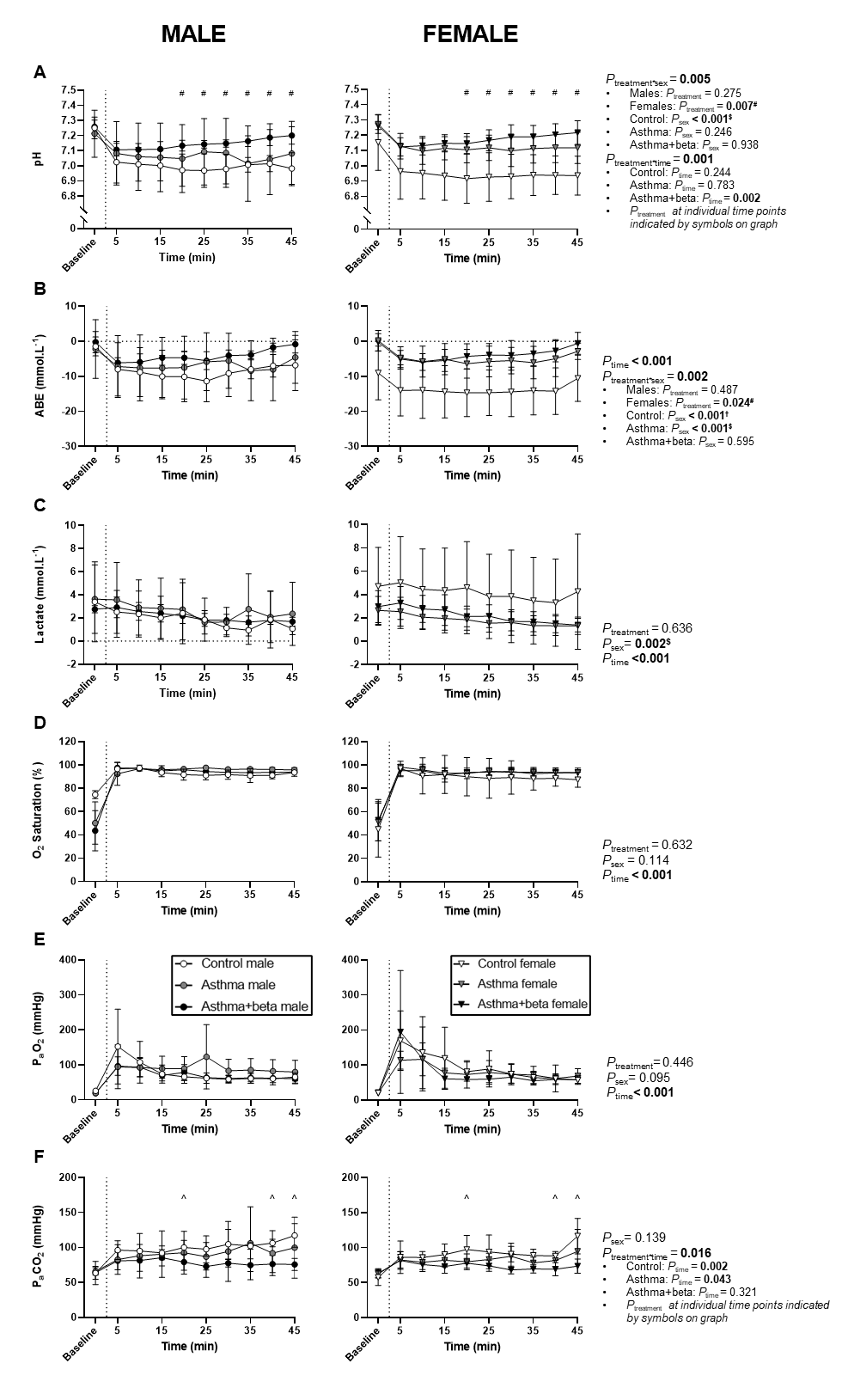
**

***Figure S1. Blood chemistry in lambs during the neonatal lung function study.*** *Data are from lambs born to control ewes (control, open shapes, male n=8, female n=7) asthmatic ewes (asthma, grey shapes, male n=7, female n=5) and lambs born to asthmatic ewes treated with antenatal betamethasone (asthma+beta, closed shapes, male n=4, female n=7). Data shows measures over time for pH (A), actual base excess (B, ABE), lactate (C), saturation of haemoglobin with oxygen (D, S_a_O_2_), partial pressure of oxygen (E, PaO_2_), and partial pressure of carbon dioxide (F, PaCO_2_) in arterial blood. Fetal data (baseline) were measured before delivery. Data are separated by sex: male lambs (circles, left panels) and female lambs (triangles, right panels), and are shown as mean ± SD at each time point. Statistical significance (P < 0.05) is shown in bold, and interactions are reported when significant. Bonferroni correction was used to determine differences between groups where an overall treatment effect was significant. ^#^control < asthma+beta. ^control > asthma+beta. ^†^males > females. ^$^males < females.*


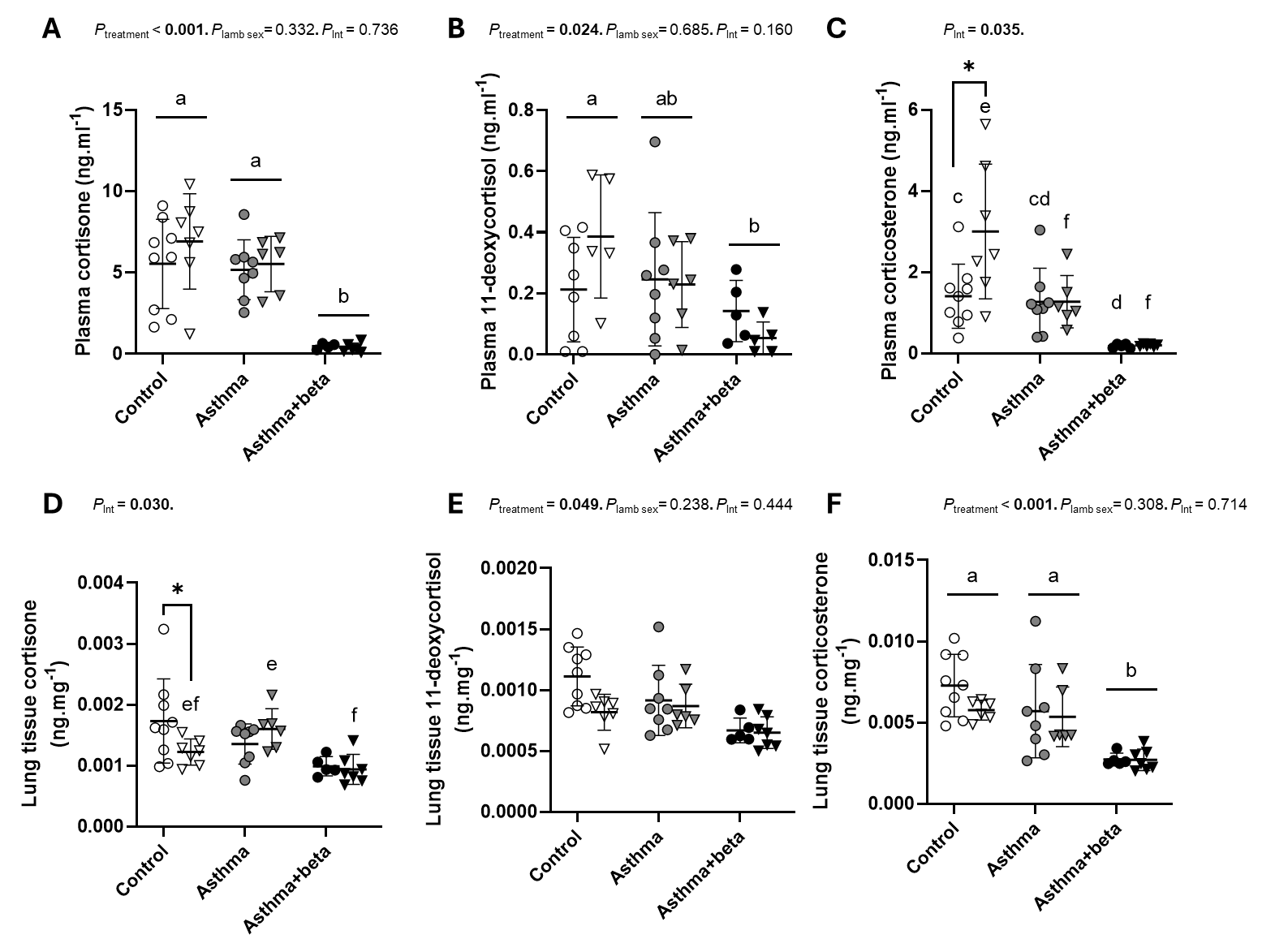


***Figure S2. Plasma and lung tissue glucocorticoid concentrations.*** *Data shows glucocorticoid concentrations in plasma (A-C) and lung tissue (D-F) for cortisone (A, D), 11-deoxycortisol (B, E), and corticosterone (C, F). Data are separated by lamb sex into males (circles) and females (triangles). Data are from* *control lambs (open shapes, male n=8-9, female n = 5-7), lambs born to asthmatic ewes (asthma, grey shapes, male n=8, female n=6), and lambs born to asthmatic ewes treated with antenatal betamethasone (asthma+beta, closed shapes, male n=4-5, female n=6-7). Each symbol indicates data from one lamb, with whisker plots showing the mean ± SD for each group. Statistical significance (P < 0.05) is shown in bold. Bonferroni correction was used to determine differences between groups where an overall treatment effect was significant, indicated by different letters. Letters indicate differences between groups overall (a, b), within males (c, d) or within females (e, f). Asterisks (*) denote the effect of sex within treatment groups. P_int_ = treatment*sex interaction analysis*

**
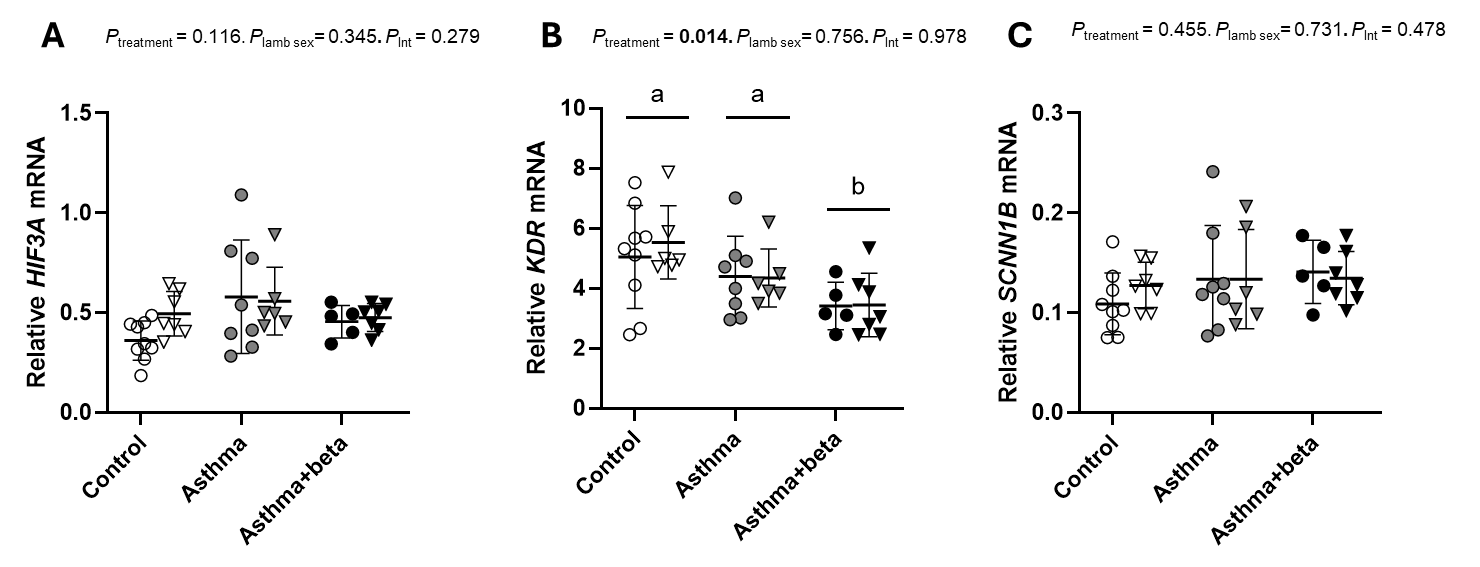
**

***Figure S3. HIF3A, KDR and SCNN1B gene expression.*** *Data shows gene expression of HIF3A (A), KDR (B) and SCNN1B (C). Data are separated by lamb sex into males (circles) and females (triangles). Data are from* *control lambs (open shapes, male n=9, female n = 6-7), lambs born to asthmatic ewes (asthma, grey shapes, male n=8, female n=6), and lambs born to asthmatic ewes treated with antenatal betamethasone (asthma+beta, closed shapes, male n=5, female n=7). Each symbol indicates data from one lamb, with whisker plots showing the mean ± SD for each group. Statistical significance (P < 0.05) is shown in bold. Bonferroni correction was used to determine differences between groups where an overall treatment effect was significant, indicated by different letters. Letters indicate differences between groups overall (a, b). P_int_ = treatment*sex interaction analysis*
