# Supplementary material for: Betamethasone improved near‐term neonatal lamb lung maturation in experimental maternal asthma
Source: Exp Physiol. 2024 Oct 22;109(11):1967–79. doi: 10.1113/EP091997 (PMC11522833; doi:10.1113/EP091997)
Supplement: Supplementary file 4 — Supporting information [file EPH-109-1967-s003.docx]

**Betamethasone improved near-term neonatal lamb lung maturation in experimental maternal asthma**

Joshua L. Robinson^1,2,3^, Andrea J. Roff^1,3,4^, Sarah J. Hammond^1,3^, Jack R. T. Darby^3^, Ashley S. Meakin^3^, Stacey L. Holman^3^, Andrew Tai^1,2,5^, Tim J. M. Moss^6^, Catherine G. Dimasi^3^, Sarah M.

Jesse^3^, Michael D. Wiese^7^, Andrew N. Davies^8^, Beverly S. Muhlhausler^9^, Robert J. Bischof^10^, Megan J. Wallace^6,11^, Vicki L. Clifton^12^, Janna L. Morrison^3^, Michael J. Stark^1,2,5^, and Kathryn L. Gatford^1,4^

**Author Affiliations**

*^1^Robinson Research Institute, University of Adelaide, Adelaide, SA 5005, Australia*

*^2^Adelaide Medical School, University of Adelaide, Adelaide, SA 5005, Australia*

*^3^Early Origins of Adult Health Research Group, Health and Biomedical Innovation, Clinical and Health Sciences, University of South Australia, Adelaide, SA 5005, Australia*

*^4^School of Biomedicine, University of Adelaide, Adelaide, SA 5005, Australia*

*^5^Women’s & Children’s Hospital, North Adelaide, SA 5006, Australia*

*^6^Department of Obstetrics and Gynaecology, Monash University, Clayton, VIC 3800, Australia*

*^7^Centre for Pharmaceutical Innovation, Clinical & Health Sciences, University of South Australia, Adelaide, SA 5005, Australia*

*^8^Biomedicine Discovery Institute, Monash University, Frankston, VIC 3199, Australia*

*^9^Commonwealth Scientific and Industrial Research Organization, Adelaide, SA 5000, Australia*

*^10^Institute of Innovation, Science, and Sustainability, Federation University Australia, Berwick, VIC 3806, Australia*

*^11^The Ritchie Centre, Hudson Institute of Medical Research, Clayton, VIC 3168, Australia*

*^12^Mater Medical Research Institute, University of Queensland, South Brisbane, QLD 4101, Australia*

**Corresponding author**

A/Prof Kathryn Gatford

Robinson Research Institute, School of Biomedicine, Level 6, Adelaide Health and Medical Sciences Building, University of Adelaide, Adelaide, SA 5005, Australia

Phone: +61 8 83134158; Email: [kathy.gatford@adelaide.edu.au](mailto:kathy.gatford@adelaide.edu.au)

**Keywords:** Disease Models, Animal; Pregnancy Complications, physiopathology; Glucocorticoids; Respiratory Distress Syndrome, Newborn; sheep

**Running title:** Antenatal betamethasone treatment in maternal asthma

**Total word count:** 3776

**Total references number:** 59

**Subject area:** Respiratory

**New Findings**

***What is the central question of this study?***

- In previous studies, maternal asthma reduced lung surfactant expression in near-term fetal sheep.
- We investigated whether antenatal betamethasone before near-term delivery may mitigate the increased risk of neonatal lung disease in a sheep model of maternal asthma.

***What is the main finding and its importance?***

- Maternal asthma did not impair neonatal lung function or reduce surfactant gene expression, likely reflecting a mild maternal and fetal phenotype in this cohort.
- Nevertheless, antenatal maternal betamethasone treatment in asthmatic pregnancies increased dynamic lung compliance and surfactant protein A and B mRNA expression in newborn lambs.
- These data suggest potential benefits of betamethasone on lung maturation when mothers have asthma.

**Abstract**

Maternal asthma is associated with increased rates of neonatal lung disease, and fetuses from asthmatic ewes have fewer surfactant-producing cells and lower surfactant-protein B gene (*SFTPB)* expression than controls. Antenatal betamethasone increases lung surfactant production in preterm babies, and we therefore tested this therapy in experimental maternal asthma. Ewes were sensitised to house dust mite allergen, and an asthmatic phenotype induced by fortnightly allergen lung challenges; controls received saline. Pregnant asthmatic ewes were randomised to receive antenatal saline (asthma) or 12 mg intramuscular betamethasone (asthma+beta) at 138 and 139 days of gestation (term = 150 days). Lambs were delivered by Caesarean section at 140 days of gestation and ventilated for 45 minutes before tissue collection. Lung function and structure were similar in control lambs (n=16, 11 ewes) and lambs from asthma ewes (n=14, 9 ewes). Dynamic lung compliance was higher in lambs from asthma+beta ewes (n=12, 8 ewes) compared to those from controls (*P* = 0.003) or asthma ewes (*P* = 0.008). Lung gene expression of surfactant proteins *SFTPA* (*P* = 0.048) and *SFTPB* (*P* < 0.001), but not *SFTPC* (*P* = 0.177) or *SFTPD* (*P* = 0.285), was higher in lambs from asthma+beta than those from asthma ewes. Female lambs had higher tidal volume (*P* = 0.007), dynamic lung compliance (*P* < 0.001), and *SFTPA* (*P* = 0.037) and *SFTPB* gene expression (*P* = 0.030) than males. This data suggests that betamethasone stimulates lung maturation and function of near-term neonates, even in the absence of impairment by maternal asthma.

**Introduction**

Asthma is a chronic condition characterised by hyperresponsiveness and inflammation of the airways that affects ~9-17% of pregnant women worldwide (Kwon *et al.*, 2006; Das *et al.*, 2021; Fujino *et al.*, 2021). Approximately 50% of pregnant women with asthma experience loss of control, involving increased symptom severity, or asthma exacerbations, leading to a need for medical intervention and increased medication use (Grzeskowiak *et al.*, 2016). Maternal asthma in pregnancy is associated with increased risks of complications including gestational diabetes, preeclampsia, preterm delivery, and Caesarean section (Murphy *et al.*, 2011; Hodyl *et al.*, 2014; Wang *et al.*, 2014; Robinson *et al.*, 2023b). Maternal asthma is also associated with increased risks of adverse neonatal outcomes including perinatal mortality, nursery admission, transient tachypnoea of the newborn (TTN), respiratory distress syndrome (RDS), low birthweight and small for gestational age (Murphy *et al.*, 2011; Murphy *et al.*, 2013; Hodyl *et al.*, 2014; Mendola *et al.*, 2014; Robinson *et al.*, 2023b), but the mechanisms are largely unknown. RDS and TTN are both associated with increased work of breathing and insufficient alveolar surfactant (Machado *et al.*, 2011; Reuter *et al.*, 2014; McGillick *et al.*, 2017). In an experimental sheep model of maternal asthma before and during pregnancy, the lungs of near-term fetuses from asthmatic ewes had fewer surfactant-producing type II alveolar epithelial cells and lower surfactant protein gene expression compared to controls (Clifton *et al.*, 2016; Wooldridge *et al.*, 2019), suggesting that maternal asthma leads to newborn surfactant-deficiency.

Antenatal corticosteroids (ACS) mature the fetal lung by increasing numbers of type II alveolar epithelial cells, increasing surfactant production, and thinning the alveolar walls (Wallace *et al.*, 1995, 1996; Polglase *et al.*, 2007). Treatment with ACS is recommended for women at risk of preterm delivery before 34-37 weeks completed gestation, depending on jurisdiction: 24 mg given in two (betamethasone) or four (dexamethasone) doses over the 48 hours prior to expected delivery (Antenatal Corticosteroid Clinical Practice Guidelines Panel, 2015; Reddy *et al.*, 2021; World Health Organization, 2022). ACS are not currently recommended for use before term delivery. However, given the evidence of lung immaturity in near-term fetal sheep from asthmatic ewes (Clifton *et al.*, 2016; Wooldridge *et al.*, 2019) and increased risk of RDS in preterm and term human neonates exposed to maternal asthma (Mendola *et al.*, 2014), we hypothesised that ACS therapy in pregnancies complicated by asthma would reduce the incidence of lung morbidities at term birth. We therefore compared neonatal lung function, lung structure, and surfactant protein gene expression in lambs from control ewes, asthmatic ewes treated with saline (asthma), and asthmatic ewes treated with antenatal betamethasone (asthma+beta). We did not use a control group treated with antenatal betamethasone given that steroids are not clinically recommended for women who are expected to deliver at term (World Health Organization, 2022); we only explored betamethasone as a potential intervention for the offspring of asthmatic mothers. We also explored the effect of lamb sex on outcomes and whether responses to treatment differed between sexes.

**Methods**

*Ethics approval*

The South Australian Health and Medical Research Institute (SAHMRI) Animal Ethics Committee approved this study (SAM455.19) according to the Australian code for the care and use of animals for scientific purposes (National Health and Medical Research Council, 2013). All investigators adhered to the ethical principles outlined by Grundy (Grundy, 2015) and the principles of the 3Rs (Tannenbaum & Bennett, 2015).

*Animals and induction of maternal asthma phenotype*

Ewes were sensitised to house dust mite (HDM) and then subjected to repeated airway challenges with HDM to induce an asthmatic phenotype (Fig. 1), following our published protocols (Bischof *et al.*, 2003; Clifton *et al.*, 2016). Control ewes received saline airway challenges. Ewe lung function was measured in late pregnancy (~132 days of gestation, dG), immediately prior to challenge with HDM. Ewe lung eosinophils were measured in bronchoalveolar lavage collected before and 48 h after this challenge. Further details on sensitisation, induction of asthma and phenotype measures are provided in the supplementary methods.

*Mating, treatments, lamb delivery and ventilation*

After 10 weeks of HDM challenges, ewes were fitted with an intravaginal controlled internal drug release device containing progesterone (Eazi-breed CIDR, Zoetis Australia Pty Ltd, Rhodes, NSW, Australia) to synchronize oestrus prior to mating with Merino rams. Fortnightly airway challenges were continued throughout the study. Pregnancy was confirmed by ultrasound at 55 dG (Fig 1). Singleton and twin-bearing asthmatic ewes were randomised using a random number generator to receive saline or betamethasone (Celestone Chronodose 11.6 mg, Schering Plough, Baulkham Hills, NSW, Australia), with intramuscular injections 48 and 24 hours prior to delivery, consistent with clinical practice (Antenatal Corticosteroid Clinical Practice Guidelines Panel, 2015). Control ewes received saline injections. A 150 mg dose of medroxyprogesterone acetate was given intramuscularly to all ewes 6-9 days prior to antenatal injections to prevent premature labour due to betamethasone-induced progesterone withdrawal (Jenkin *et al.*, 1985; Jobe *et al.*, 2003). Lambs were delivered by Caesarean section at 140 dG and ventilated for 45 minutes with a volume guarantee strategy, as detailed in supplementary methods. After ventilation, the lambs were humanely killed with sodium pentobarbitone (20 mg.kg^-1^_­_, Virbac Australia, Peakhurst, NSW, Australia). The left lung was removed, weighed, and inflation fixed (20 cmH_2_0) in 4% paraformaldehyde. Samples from the fixed left cranial and left caudal lobes of each lung were paraffin-embedded and sectioned (4 µm), and sections from the right caudal lobe were frozen in liquid nitrogen (Westover *et al.*, 2012). Additional lamb tissues were extensively sampled consistent with best practice (Morrison *et al.*, 2018).

*Neonatal Lung Maturation*

Tissue-to-airspace ratio and type II alveolar epithelial cell density were determined in caudal and cranial lung sections from each lamb (Lock *et al.*, 2015). Surfactant protein gene expression was determined by quantitative real-time PCR (Orgeig *et al.*, 2010; McGillick *et al.*, 2013; Lock *et al.*, 2017). Concentrations of glucocorticoids (cortisol, cortisone, 11-deoxycortisol, and corticosterone) in arterial plasma prior to delivery and in lung tissue collected at the end of the study were determined by mass spectrophotometry (Dimasi *et al.*, 2023; Lock *et al.*, 2023). These analyses are described in more detail in the supplementary methods.

*Statistical Analyses*

Maternal lung function and immune responses to treatment, collected before allocating asthmatic ewes to saline or betamethasone treatment, were compared between control and asthmatic ewes by 1-way ANOVA. Repeated measures ANOVA was used to compare the proportion of eosinophils in BAL before and after the airway challenge. One fetus (control female) died *in utero*, resulting in the delivery of a total of 42 lambs for ventilation and tissue collection. Four lambs (1 control male, 1 asthma male, 1 asthma female, and 1 asthma+beta male) were excluded from functional analyses due to changes in the position and ventilation strategy during the initial optimization of the protocol. Four lambs (1 control male, 2 asthma females, 1 asthma+beta female) were excluded from tissue-to-airspace ratio analyses due to poor fixation of the lung. Any missing blood gas or ventilation data for the remaining animals was due to machine calibrations. Lamb glucocorticoid and lung structural outcomes were analysed with treatment and lamb sex, plus lung region as a repeated measure, with ewe as a random factor to correct for the effects of maternal environment. Singleton (n = 13) and twin (n = 15) pregnancies were included resulting in 43 lambs. In preliminary analyses, litter size had minimal effect on outcomes, and, therefore, we prioritised lamb sex for inclusion in statistical analysis; there were insufficient animal numbers to include both factors. Data collected during the lamb ventilation protocol was analysed by linear mixed model using treatment and lamb sex as factors, plus time as a repeated measure, and ewe as a random factor to correct for the effects of maternal environment. A Bonferroni correction was used to compare groups where there was an overall effect of ewe treatment. Where interactions were significant, further subgroup analyses were performed. Data were analysed using SPSS version 27 (IBM Corporation, Armonk, New York, USA). If *P <* 0.05 the null hypothesis was rejected. Data are presented as mean (standard deviation, SD).

**Results**

*Ewe and lamb phenotype*

In late pregnancy (~132 dG) there was no difference in pre-challenge dynamic lung compliance between saline (0.0367 (0.0130) L.cm-1 H_2_O) and asthma ewes (0.0333 (0.0140) L.cm-1 H_2_O; P = 0.529). Although we did not see an effect of asthma on lung function within the pregnant animals included in the present manuscript, dynamic lung compliance decreased more in asthmatic than control ewes between premating and ~132 dG in analyses of the whole cohort including non-pregnant ewes (*P* = 0.040)*.* Eosinophil concentrations in BAL were not different between control and asthma ewes either pre- (12690 (18957) n.ml-1 cf. 6770 (10319) n.ml-1; *P* = 0.294) or post-challenge (91486 (279191) cf. 26808 (53339) n.ml-1; P = 0.357). Eosinophil concentrations were higher 48 hours post-challenge than pre-challenge in both control (*P =* 0.045) and asthma groups (*P* = 0.008).

At 140 dG, there was an overall effect of treatment on ewe weight (*P* = 0.037, Table 1) but in pairwise comparisons body weight was not different between control ewes and asthma (*P* = 0.164) or asthma+beta ewes (*P* = 0.051), nor between asthma and asthma+beta ewes (*P* = 1.000). When comparing all groups, treatment and lamb sex did not affect absolute or relative lamb body weight at delivery, nor absolute or relative weights of lamb lungs or brains (Table 1). In additional analyses combining all lambs from asthmatic ewes, absolute birth weight did not differ between lambs from control and asthmatic ewes (*P* = 0.523), but birth weight relative to maternal weight was 12.5% lower in lambs of asthmatic ewes compared to those born to control ewes (*P* = 0.046).

*Ventilation parameters*

The effects of treatment on tidal volume relative to lamb weight (Fig 2A) differed with lamb sex (interaction, *P* = 0.002) and relative tidal volume increased over time (*P* = 0.048). Treatment affected relative tidal volume in male (*P* = 0.036) but not female lambs (*P* = 0.203). In males, relative tidal volume was lower in control than asthma+beta lambs (*P* = 0.044), and not different in asthma and either asthma+beta (*P* = 0.103) or control lambs (*P* = 1.000). Relative tidal volume did not differ between sexes within control (*P* = 0.221) or asthma+beta groups (*P* = 0.345) but was higher in females than males within asthma lambs (*P* < 0.001). The fraction of inspired oxygen required during ventilation (FiO_2_, Fig 2B) did not differ between treatments (*P* = 0.056; Fig 2B) or sexes (*P* = 0.151), and decreased over time (*P* < 0.001). Minute ventilation (Fig 2C) differed with treatment (*P* = 0.022) and sex (males < females; *P* < 0.001) and did not change over time (*P* = 0.971). Minute ventilation was lower in asthma than control lambs (*P* = 0.019) but did not differ between asthma+beta lambs and either control (*P* = 0.353) or asthma (*P* = 0.670) lambs. Dynamic lung compliance (Fig 2D) differed with treatment (*P* = 0.002) and sex (males < females; *P* < 0.001) and increased over time (*P* < 0.001). Compliance was higher in asthma+beta lambs than in either control (*P* = 0.003) or asthma (*P* = 0.008) lambs with no difference between lambs from asthma and control groups (*P* = 1.000). Raw data for Figure 2 are available in the supporting information.

*Physiological parameters*

The effects of treatment on ETCO_2_ (Fig 3A) differed with lamb sex (interaction, *P* < 0.001) and time (interaction, *P* < 0.001). There was an effect of treatment on ETCO_2_ within males (*P* = 0.033) and females (*P* = 0.046). In males, asthma+beta had higher ETCO_2_ than control lambs (*P* = 0.031), but ETCO_2_ of asthma lambs did not differ from asthma+beta (*P* = 0.392) or controls (*P* = 0.597). In females, ETCO_2_ did not differ between control and asthma+beta (*P* = 0.114), control and asthma (*P* = 0.071), or asthma and asthma+beta groups (*P* = 1.000). ETCO_2_ did not differ between sexes in control (*P* = 0.842) or asthma lambs (*P* = 0.294) but was higher in males than females within asthma+beta lambs (*P* < 0.001). ETCO_2_ increased over time in control (*P* < 0.001) and asthma lambs (*P* = 0.006) but did not change with time in asthma+beta lambs (*P* = 0.122). Asthma+beta lambs had higher ETCO_2_ than control lambs from 5 until 30 minutes of the ventilation protocol, and higher ETCO_2_ than asthma lambs at 5 and 10 minutes (all *P* < 0.05); ETCO_2_ did not differ between control and asthma lambs at any time point. Heart rate (Fig 3B) differed between treatments (*P* = 0.002) and sexes (male > female, *P* < 0.001), and increased over time (*P* = 0.013). Heart rate was higher in asthma+beta lambs than in control (*P* = 0.007) or asthma lambs (*P* = 0.006) but was not different in asthma and control lambs (*P* = 1.000). Arterial partial pressures of oxygen and carbon dioxide are detailed with other blood biochemistry measures in the supplementary results (Fig S1). Raw data for Figure 3 and S1 are available in the supporting information.

Cortisol concentrations in plasma (Fig 4A) differed with treatment (*P* < 0.001). Plasma cortisol concentrations were lower in asthma+beta lambs compared to control (*P* < 0.001) or asthma lambs (*P* < 0.001), and did not differ between lambs from asthma ewes and control ewes (*P* = 0.308). Cortisol concentrations in lung tissue collected at the end of the study (Fig 4B) differed with treatment (*P* < 0.001). Lung tissue cortisol concentrations were lower in asthma+beta lambs compared to control (*P* < 0.001) or asthma lambs (*P* < 0.001), and did not differ between lambs from asthma ewes and control ewes (*P =* 1.000 respectively). Neither did cortisol concentrations differ between male and female lambs (*P* = 0.074 and *P* = 0.781 respectively*,* Fig 4). Antenatal betamethasone treatment similarly suppressed plasma cortisone, 11-deoxycortisol and corticosterone concentrations and suppressed lung tissue corticosterone concentrations.

*Lung structure and surfactant protein mRNA expression*

Tissue-to-airspace ratio (Fig 5D) was not affected by treatment (*P* = 0.260) or sex (*P* = 0.406). The density of type II cells (Fig 5H) was similarly unaffected by treatment (*P* = 0.101) or sex (*P* = 0.244). Expression of *SFTPA* (Fig 5I) and *SFTPB* (Fig 5J) differed with treatment (*P* = 0.005 and *P* < 0.001 respectively) and sex (females > males; *P* = 0.037 and *P* = 0.030 respectively). Expression of *SFTPA* and *SFTPB* was higher in asthma+beta than control (*P* = 0.004 and *P* < 0.001 respectively) or asthma lambs (*P* = 0.048 and *P* < 0.001 respectively) and did not differ between asthma and control lambs (*P* = 1.000 and *P* = 0.895 respectively). Expression of *SFTPC* (Fig 5K) differed with treatment (*P* = 0.006) but not sex (*P* = 0.199). Expression of *SFTPC* was higher in asthma+beta than control lambs (*P* = 0.005), and similar in asthma and control lambs (*P* = 0.381) or asthma+beta lambs (*P* = 0.177). Expression of *SFTPD* (Fig 5L) was unaffected by treatment (*P* = 0.241) or sex (*P* = 0.322). Gene expression of *HIF3A* AND *SCNN1B* did not differ with treatment or sex, whilst gene expression of *KDR* differed with treatment but not sex (supplementary results, Fig S3).

**Discussion**

The results of this study support our hypothesis that antenatal betamethasone may improve term neonatal lung function in the offspring of asthmatic mothers, in the context of a mild maternal phenotype. Betamethasone treatment increased dynamic lung compliance and *SFTPA* and *SFTPB* gene expression in lambs of asthmatic ewes compared to lambs from both control and untreated asthma ewes. Female lambs had higher dynamic lung compliance and expression of *SFTPA* and *SFTPB* than males. While maternal asthma did not impair neonatal lung function or surfactant gene expression in this study, our findings demonstrate that, even near term, antenatal betamethasone improves neonatal lung function, particularly in male offspring.

The lack of impact of maternal asthma on lamb surfactant gene expression differs from our previous study employing this model (Clifton *et al.*, 2016), which may reflect a milder maternal asthma phenotype in the present cohort. In this cohort, lung function and BAL eosinophil count in ewes, and lung compliance and type II cell density in near-term lambs, did not differ between control and asthma groups. Nevertheless, in the larger cohort of animals that included non-pregnant ewes, dynamic lung function decreased more in asthmatic than control ewes between mating and ~132 dG, indicating mild maternal asthma. The milder phenotype in these ewes differs from previous cohorts of sheep in which the same protocol was used to induce asthma, including in the context of pregnancy (Bischof *et al.*, 2003; Clifton *et al.*, 2016; Wooldridge *et al.*, 2019). Neonatal outcomes, including fetal growth (Stevens *et al.*, 2023), rates of preterm birth, nursery admission, and congenital malformations, are improved by better control and less severe asthma in humans (Abdullah *et al.*, 2020; Yland *et al.*, 2020), and thus the milder asthma phenotype in the present study is likely to have less impact on neonates. Despite this, relative lamb birth weight was lower in progeny of asthmatic ewes compared to those from control ewes irrespective of betamethasone treatment, by a similar magnitude (-12.5%) as in our previous study in which ewes developed a more severe asthma phenotype (Clifton *et al.*, 2016). Although ewes in both the current and previous studies were all Merinos, the South Australian strain from the present study were ~2-fold heavier than the Victorian strain in the original study, resulting in lower relative lung exposure to HDM, as the same dose was used (Clifton *et al.*, 2016). The different ewe phenotype may also reflect differences in immune function between animal cohorts, possibly reflecting prior or current pathogen and environmental exposures, or HDM batch differences. Given that impacts of maternal asthma depend on severity, benefits of antenatal betamethasone are likely to be greater in the context of poorly controlled or severe maternal asthma, and decisions to treat should consider the maternal phenotype.

In lambs delivered near-term, betamethasone treatment increased dynamic lung compliance and elevated *SFTPA* and *SFTPB* expression relative to saline-treated asthma lambs and increased dynamic lung compliance and elevated *SFTPA*, *SFTPB*, and *SFTPC* gene expression relative to controls. The hydrophobic *SFTPB* and *SFTPC* are the two surfactant proteins primarily responsible for reducing surface tension (Haagsman & Diemel, 2001), and increases in their expression likely contribute to better dynamic lung compliance in the betamethasone-treated group. This upregulation of surfactant protein expression by antenatal betamethasone is similar to effects reported at earlier gestational ages. Antenatal betamethasone induced elevated *SFTPA* and *SFTPD* gene expression in lambs born at 96 dG (Visconti *et al.*, 2018), while the gene expression of all four surfactant proteins (A to D) was increased at ~124 dG in response to betamethasone treatment 24 and 48 hours earlier (Schmidt *et al.*, 2017). Betamethasone did not alter type II epithelial cell density in this cohort, consistent with findings in 125 dG sheep (Visconti *et al.*, 2018). However, other responses to betamethasone differ between our study and previous reports, which may be due to the late gestational age of our lambs (140 dG; term ~150 dG). The lack of betamethasone-induced changes in lamb lung structure in the present study contrasts with reported increases in proportion of airspace and decreases in tissue at 124 dG (Polglase *et al.*, 2007).The endogenous plasma cortisol surge commences at ~134-137 dG in sheep (Phillips *et al.*, 1996; Orgeig *et al.*, 2010), which may have induced structural lung maturation before our antenatal treatments. Also contrasting with results of other studies, and potentially reflecting differences in gestational age, time from exposure or species, *HIF3A*, *KDR*, and *SCNN1B* were not elevated in betamethasone-exposed lambs compared to unexposed lambs in the present study, although betamethasone upregulates these genes in near-term fetal rat lung fibroblasts six hours after exposure and 94 dG sheep fetuses 48 hours after exposure (Visconti *et al.*, 2018; Seow *et al.*, 2019).

A striking observation in the present study was the lower abundance of endogenous glucocorticoids in plasma and lung tissue in betamethasone-exposed lambs, likely reflecting negative feedback of glucocorticoid receptor activation on endogenous cortisol production. Total circulating glucocorticoid activity, assessed first by radioreceptor assays and later by systems using cells transfected with reporter genes downstream of the human glucocorticoid receptor gene, is increased within 12 hours and remains elevated for 1-5 days following maternal antenatal betamethasone treatment (Ballard *et al.*, 1980; Kajantie *et al.*, 2004). This increase reflects the activity of the exogenous steroid, since cortisol and DHEA concentrations in cord blood are suppressed within 1-6 hours of maternal betamethasone injection to concentrations less than half those of infants who were untreated or last exposed more than 7 d previously (Ballard *et al.*, 1975; Ballard *et al.*, 1980; Parker *et al.*, 1996; Kajantie *et al.*, 2004; Süvari *et al.*, 2021). Circulating cortisol remains suppressed for up to 7 days after antenatal betamethasone (Ballard *et al.*, 1980; Parker *et al.*, 1996; Kajantie *et al.*, 2004). Less data is available on effects of antenatal dexamethasone on endogenous steroid production, although serum cortisol concentrations measured within 12 h of birth were ~30% lower in neonates whose mothers had been treated with antenatal dexamethasone within 7 days of delivery (Karlsson *et al.*, 2000). More detailed pharmacokinetic studies have recently been reported in women of reproductive age, whose plasma cortisol concentrations were suppressed until 60 hours after a single 6 mg.mL^-1^ intramuscular dose of betamethasone (Jobe *et al.*, 2020). This suggests that exposure to antenatal betamethasone suppresses endogenous cortisol secretion for similar durations in the fetus, where concentrations can only be measured at delivery, as in adult women. Using LC-MS/MS methodology and avoiding potential issues of steroid cross-reactivity in other assays, we have demonstrated even more profound suppression of circulating and lung cortisol concentrations in preterm lambs at 24 h after the second dose of antenatal betamethasone. Furthermore, we have extended these findings to also demonstrate that a single course of maternal antenatal betamethasone also suppresses concentrations of cortisol in fetal lung tissue, and reduces fetal circulating concentrations of additional endogenous steroids.

In the present study, male lambs had poorer respiratory outcomes and evidence of lung immaturity relative to females, with lower actual tidal volume, dynamic lung compliance, and *SFTPA* and *SFTPB* gene expression, independent of treatment. If also present in humans, these sex differences may contribute to the greater risk of neonatal respiratory morbidity and poorer childhood lung function in preterm-born males compared to preterm-born females (Laube & Thome, 2022). Endocrine differences may underlie sexual dimorphism in normal lung maturation, with evidence in piglets that oestrogen and progesterone promote alveolar formation (Trotter *et al.*, 2006), and studies in rabbits suggesting that androgens may inhibit fetal lung production of surfactants (Nielsen *et al.*, 1982).

Our data also support the hypothesis that responses to ACS differ between sexes. Relative tidal volume and ETCO_2_ were higher in asthma+beta compared to control males but did not differ between asthma+beta and control females. Conversely, arterial pH and ABE were higher in asthma+beta compared to control females but did not differ between asthma+beta and control males. In the present study, effects of betamethasone on lung compliance were similar in male and female lambs delivered at 140 dG, in contrast to a study of 128 dG preterm lambs, where benefits of betamethasone on lung compliance were greater in females than males (Willet *et al.*, 1997). Overall, the smaller benefits in females in the present study likely reflect greater maturation of the female lung in the near-term sheep, as discussed above, lessening the opportunity for further responses to exogenous corticosteroids. Clinically, evidence for sex differences in response to ACS is mixed, with improved survival and reduced rates of intubation and incidence of bronchopulmonary dysplasia in preterm (<29 weeks gestation) males but not females in one study (Ramos-Navarro *et al.*, 2022), but reduced the risk of RDS in preterm (28-33 weeks) females but not males in another study (Collaborative Group on Antenatal Steroid Therapy, 1981). Additional comparisons between sexes across gestational ages are needed to determine sex differences in response in humans.

Strengths of the study include using sheep as a preclinical model of pregnancy complications and for testing antenatal interventions, since the maturity of the sheep lung at birth is more comparable to the lungs of humans than rats or mice (Lock *et al.*, 2013; Morrison *et al.*, 2018). Maternal asthma was induced using a well-established protocol (Bischof *et al.*, 2003; Clifton *et al.*, 2016; Wooldridge *et al.*, 2019) and key characteristics of asthma phenotype were measured (Robinson *et al.*, 2023a), although the maternal asthma phenotype was milder than previously reported (Clifton *et al.*, 2016). Limitations of this study include potential lack of statistical power to detect differences, since not every treatment group contained seven lambs per sex, which we calculated would be required for to detect a 20% change (at 80% power) in surfactant gene expression and alveolar cells densities based on our previous studies (Clifton *et al.*, 2016; Wooldridge *et al.*, 2019). Nevertheless, effects of antenatal betamethasone were clearly evident. An additional limitation of the study includes the unexpectedly high proportion of twin pregnancies, which introduced an additional source of fetal growth restriction (Muhlhausler *et al.*, 2011) and thus may have reduced the ability to detect structural and functional lung differences, in contrast to our previous singleton-only study (Clifton *et al.*, 2016). Additionally, some baseline lung ventilation parameters in the controls differed from those previously reported in studies of ventilated lambs of a similar gestational age (Schmölzer *et al.*, 2022; Yamaoka *et al.*, 2022), with greater acidosis, hypercapnia, and oxygen demand. We did not include a group of control ewes treated antenatally with betamethasone in the present study since we were evaluating an intervention to improve neonatal respiratory outcomes in the setting of maternal asthma and antenatal betamethasone is not currently clinically recommended for use in women expected to deliver at term (Reddy *et al.*, 2021).

An improvement in neonatal dynamic lung compliance, induced by betamethasone treatment of asthmatic mothers, may reduce the risk of lung disease at birth, particularly in males. We suggest that the long-term consequences of betamethasone should also be studied to determine if any benefit for newborn lung function is sustained given that adverse respiratory outcomes associated with maternal asthma are also seen in childhood (Lim *et al.*, 2010; Berry *et al.*, 2016). At present, ACS are clinically advised only before 37 weeks of gestation, where they benefit lung maturation and short-term morbidity (Malaeb & Stonestreet, 2014; Reddy *et al.*, 2021). Before ACS use is extended to term births in mothers with asthma, we also suggest that long-term neurological outcomes should be investigated given concerns regarding the adverse effects of ACS treatment on childhood neurodevelopment (Ninan *et al.*, 2022; Vidaeff *et al.*, 2023). Our data also provides evidence that mild maternal asthma does not impair neonatal lung function. This is consistent with clinical evidence that good control of maternal asthma during pregnancy improves offspring respiratory health (Martel *et al.*, 2009; Collier *et al.*, 2013; Liu *et al.*, 2018; Morten *et al.*, 2018). Together, this suggests that improving maternal asthma control during pregnancy should be a priority, while antenatal betamethasone may be an effective rescue strategy to improve neonatal respiratory health where maternal asthma is not well-controlled.

**References**

Abdullah K, Zhu J, Gershon A, Dell S & To T. (2020). Effect of asthma exacerbation during pregnancy in women with asthma: a population-based cohort study. *Eur Respir J* **55,** 1901335.

Antenatal Corticosteroid Clinical Practice Guidelines Panel. (2015). Antenatal corticosteroids given to women prior to birth to improve fetal, infant, child and adult health: clinical practice guidelines. Liggins Institute Auckland, New Zealand.

Ballard PL, Gluckman PD, Liggins GC, Kaplan SL & Grumbach MM. (1980). Steroid and growth hormone levels in premature infants after prenatal betamethasone therapy to prevent respiratory distress syndrome. *Pediatr Res* **14,** 122-127.

Ballard PL, Granberg P & Ballard RA. (1975). Glucocorticoid levels in maternal and cord serum after prenatal betamethasone therapy to prevent respiratory distress syndrome. *J Clin Invest* **56,** 1548-1554.

Berry CE, Billheimer D, Jenkins IC, Lu ZJ, Stern DA, Gerald LB, Carr TF, Guerra S, Morgan WJ, Wright AL & Martinez FD. (2016). A distinct low lung function trajectory from childhood to the fourth decade of life. *Am J Respir Crit Care Med* **194,** 607-612.

Bischof RJ, Snibson K, Shaw R & Meeusen EN. (2003). Induction of allergic inflammation in the lungs of sensitized sheep after local challenge with house dust mite. *Clin Exp Allergy* **33,** 367-375.

Clifton VL, Moss TJ, Wooldridge AL, Gatford KL, Liravi B, Kim D, Muhlhausler BS, Morrison JL, Davies A, De Matteo R, Wallace MJ & Bischof RJ. (2016). Development of an experimental model of maternal allergic asthma during pregnancy. *J Physiol* **594,** 1311-1325.

Collaborative Group on Antenatal Steroid Therapy. (1981). Effect of antenatal dexamethasone administration on the prevention of respiratory distress syndrome. *Am J Obstet Gynecol* **141,** 276-287.

Collier CH, Risnes K, Norwitz ER, Bracken MB & Illuzzi JL. (2013). Maternal infection in pregnancy and risk of asthma in offspring. *Matern Child Health J* **17,** 1940-1950.

Crudo A, Petropoulos S, Suderman M, Moisiadis VG, Kostaki A, Hallett M, Szyf M & Matthews SG. (2013). Effects of antenatal synthetic glucocorticoid on glucocorticoid receptor binding, DNA methylation, and genome-wide mRNA levels in the fetal male hippocampus. *Endocrinology* **154,** 4170-4181.

Das J, Andrews C, Flenady V & Clifton VL. (2021). Maternal asthma during pregnancy and extremes of body mass index increase the risk of perinatal mortality: a retrospective cohort study. *J Asthma* **59,** 2108-2116.

Dimasi CG, Darby JRT, Cho SKS, Saini BS, Holman SL, Meakin AS, Wiese MD, Macgowan CK, Seed M & Morrison JL. (2023). Reduced in utero substrate supply decreases mitochondrial abundance and alters the expression of metabolic signalling molecules in the fetal sheep heart. *J Physiol***,** Advance online publication. <https://doi.org/10.1113/JP285572>.

Fujino T, Hasunuma H, Okuda M, Saito M, Utsunomiya T, Taniguchi Y, Taniguchi N, Shima M & Takeshima Y. (2021). Association between house renovation during pregnancy and wheezing in the first year of life: The Japan environment and children's study. *Allergol Int* **70,** 439-444.

Grundy D. (2015). Principles and standards for reporting animal experiments in The Journal of Physiology and Experimental Physiology. *J Physiol* **593,** 2547-2549.

Grzeskowiak LE, Smith B, Roy A, Dekker GA & Clifton VL. (2016). Patterns, predictors and outcomes of asthma control and exacerbations during pregnancy: a prospective cohort study. *ERJ Open Res* **2,** 00054-02015.

Haagsman HP & Diemel RV. (2001). Surfactant-associated proteins: functions and structural variation. *Comp Biochem Physiol A Mol Integr Physiol* **129,** 91-108.

Hodyl NA, Stark MJ, Scheil W, Grzeskowiak LE & Clifton VL. (2014). Perinatal outcomes following maternal asthma and cigarette smoking during pregnancy. *Eur Respir J* **43,** 704-716.

Jenkin G, Jorgensen G, Thorburn GD, Buster JE & Nathanielsz PW. (1985). Induction of premature delivery in sheep following infusion of cortisol to the fetus. I. The effect of maternal administration of progestagens. *Can J Physiol Pharmacol* **63,** 500-508.

Jobe AH, Milad MA, Peppard T & Jusko WJ. (2020). Pharmacokinetics and Pharmacodynamics of Intramuscular and Oral Betamethasone and Dexamethasone in Reproductive Age Women in India. *Clin Transl Sci* **13,** 391-399.

Jobe AH, Newnham JP, Moss TJ & Ikegami M. (2003). Differential effects of maternal betamethasone and cortisol on lung maturation and growth in fetal sheep. *Am J Obstet Gynecol* **188,** 22-28.

Kajantie E, Raivio T, Jänne OA, Hovi P, Dunkel L & Andersson S. (2004). Circulating glucocorticoid bioactivity in the preterm newborn after antenatal betamethasone treatment. *J Clin Endocrinol Metab* **89,** 3999-4003.

Karlsson R, Kallio J, Toppari J, Scheinin M & Kero P. (2000). Antenatal and early postnatal dexamethasone treatment decreases cortisol secretion in preterm infants. *Horm Res* **53,** 170-176.

Kwon HL, Triche EW, Belanger K & Bracken MB. (2006). The epidemiology of asthma during pregnancy: prevalence, diagnosis, and symptoms. *Immunol Allergy Clin North Am* **26,** 29-62.

Laube M & Thome UH. (2022). Y it matters - sex differences in fetal lung development. *Biomolecules* **12,** 437.

Lim RH, Kobzik L & Dahl M. (2010). Risk for asthma in offspring of asthmatic mothers versus fathers: a meta-analysis. *PLoS One* **5,** e10134.

Liu X, Agerbo E, Schlünssen V, Wright RJ, Li J & Munk-Olsen T. (2018). Maternal asthma severity and control during pregnancy and risk of offspring asthma. *J Allergy Clin Immunol* **141,** 886-892.e883.

Lock M, McGillick EV, Orgeig S, McMillen IC & Morrison JL. (2013). Regulation of fetal lung development in response to maternal overnutrition. *Clin Exp Pharmacol Physiol* **40,** 803-816.

Lock MC, Botting KJ, Allison BJ, Niu Y, Ford SG, Murphy MP, Orgeig S, Giussani DA & Morrison JL. (2023). MitoQ as an antenatal antioxidant treatment improves markers of lung maturation in healthy and hypoxic pregnancy. *J Physiol* **601,** 3647-3665.

Lock MC, McGillick EV, Orgeig S, McMillen IC, Mühlhäusler BS, Zhang S & Morrison JL. (2017). Differential effects of late gestation maternal overnutrition on the regulation of surfactant maturation in fetal and postnatal life. *J Physiol* **595,** 6635-6652.

Lock MC, McGillick EV, Orgeig S, Zhang S, McMillen IC & Morrison JL. (2015). Mature surfactant protein-B expression by immunohistochemistry as a marker for surfactant system development in the fetal sheep lung. *J Histochem Cytochem* **63,** 866-878.

Machado LU, Fiori HH, Baldisserotto M, Ramos Garcia PC, Vieira AC & Fiori RM. (2011). Surfactant deficiency in transient tachypnea of the newborn. *J Pediatr* **159,** 750-754.

Malaeb SN & Stonestreet BS. (2014). Steroids and injury to the developing brain: net harm or net benefit? *Clin Perinatol* **41,** 191-208.

Martel MJ, Rey E, Beauchesne MF, Malo JL, Perreault S, Forget A & Blais L. (2009). Control and severity of asthma during pregnancy are associated with asthma incidence in offspring: two-stage case-control study. *Eur Respir J* **34,** 579-587.

McGillick EV, Lee K, Yamaoka S, Te Pas AB, Crossley KJ, Wallace MJ, Kitchen MJ, Lewis RA, Kerr LT, DeKoninck P, Dekker J, Thio M, McDougall ARA & Hooper SB. (2017). Elevated airway liquid volumes at birth: a potential cause of transient tachypnea of the newborn. *J Appl Physiol (1985)* **123,** 1204-1213.

McGillick EV, Orgeig S, McMillen IC & Morrison JL. (2013). The fetal sheep lung does not respond to cortisol infusion during the late canalicular phase of development. *Physiol Rep* **1,** e00130.

Mendola P, Männistö TI, Leishear K, Reddy UM, Chen Z & Laughon SK. (2014). Neonatal health of infants born to mothers with asthma. *J Allergy Clin Immunol* **133,** 85-90.e81-84.

Morrison JL, Berry MJ, Botting KJ, Darby JRT, Frasch MG, Gatford KL, Giussani DA, Gray CL, Harding R, Herrera EA, Kemp MW, Lock MC, McMillen IC, Moss TJ, Musk GC, Oliver MH, Regnault TRH, Roberts CT, Soo JY, . . . Tellam RL. (2018). Improving pregnancy outcomes in humans through studies in sheep. *Am J Physiol Regul Integr Comp Physiol* **315,** R1123-R1153.

Morten M, Collison A, Murphy VE, Barker D, Oldmeadow C, Attia J, Meredith J, Powell H, Robinson PD, Sly PD, Gibson PG & Mattes J. (2018). Managing Asthma in Pregnancy (MAP) trial: FENO levels and childhood asthma. *J Allergy Clin Immunol* **142,** 1765-1772.e1764.

Muhlhausler BS, Hancock SN, Bloomfield FH & Harding R. (2011). Are twins growth restricted? *Pediatr Res* **70,** 117-122.

Murphy VE, Namazy JA, Powell H, Schatz M, Chambers C, Attia J & Gibson PG. (2011). A meta-analysis of adverse perinatal outcomes in women with asthma. *Brit J Obstet Gynaecol* **118,** 1314-1323.

Murphy VE, Wang G, Namazy JA, Powell H, Gibson PG, Chambers C & Schatz M. (2013). The risk of congenital malformations, perinatal mortality and neonatal hospitalisation among pregnant women with asthma: A systematic review and meta-analysis. *Brit J Obstet Gynaecol* **120,** 812-822.

National Health and Medical Research Council. (2013). Australian code for the care and use of animals for scientific purposes, 8th edition National Health and Medical Research Council, Canberra.

Nielsen HC, Zinman HM & Torday JS. (1982). Dihydrotestosterone inhibits fetal rabbit pulmonary surfactant production. *J Clin Invest* **69,** 611-616.

Ninan K, Liyanage SK, Murphy KE, Asztalos EV & McDonald SD. (2022). Evaluation of long-term outcomes associated with preterm exposure to antenatal corticosteroids: a systematic review and meta-analysis. *JAMA Pediatr* **176,** e220483.

Orgeig S, Crittenden TA, Marchant C, McMillen IC & Morrison JL. (2010). Intrauterine growth restriction delays surfactant protein maturation in the sheep fetus. *Am J Physiol Lung Cell Mol Physiol* **298,** L575-583.

Parker CR, Jr., Atkinson MW, Owen J & Andrews WW. (1996). Dynamics of the fetal adrenal, cholesterol, and apolipoprotein B responses to antenatal betamethasone therapy. *Am J Obstet Gynecol* **174,** 562-565.

Phillips ID, Simonetta G, Owens JA, Robinson JS, Clarke IJ & McMillen IC. (1996). Placental restriction alters the functional development of the pituitary-adrenal axis in the sheep fetus during late gestation. *Pediatr Res* **40,** 861-866.

Polglase GR, Nitsos I, Jobe AH, Newnham JP & Moss TJ. (2007). Maternal and intra-amniotic corticosteroid effects on lung morphometry in preterm lambs. *Pediatr Res* **62,** 32-36.

Ramos-Navarro C, Sánchez-Luna M, Zeballos-Sarrato S & Pescador-Chamorro I. (2022). Antenatal corticosteroids and the influence of sex on morbidity and mortality of preterm infants. *J Matern Fetal Neonatal Med* **35,** 3438-3445.

Reddy UM, Deshmukh U, Dude A, Harper L & Osmundson SS. (2021). Society for Maternal-Fetal Medicine Consult Series #58: Use of antenatal corticosteroids for individuals at risk for late preterm delivery: Replaces SMFM Statement #4, Implementation of the use of antenatal corticosteroids in the late preterm birth period in women at risk for preterm delivery, August 2016. *Am J Obstet Gynecol* **225,** B36-b42.

Reuter S, Moser C & Baack M. (2014). Respiratory distress in the newborn. *Pediatr Rev* **35,** 417-429.

Robinson JL, Gatford KL, Bailey DN, Roff AJ, Clifton VL, Morrison JL & Stark MJ. (2023a). Preclinical models of maternal asthma and progeny outcomes: a scoping review. *Eur Respir Rev***,** Accepted 09 December 2023.

Robinson JL, Gatford KL, Hurst CP, Clifton VL, Morrison JL & Stark MJ. (2023b). Do improvements in clinical practice guidelines alter pregnancy outcomes in asthmatic women? A single-center retrospective cohort study. *J Asthma* **60,** 1907-1917.

Schmidt AF, Kemp MW, Kannan PS, Kramer BW, Newnham JP, Kallapur SG & Jobe AH. (2017). Antenatal dexamethasone vs. betamethasone dosing for lung maturation in fetal sheep. *Pediatr Res* **81,** 496-503.

Schmölzer GM, Roberts CT, Blank DA, Badurdeen S, Miller SL, Crossley KJ, Stojanovska V, Galinsky R, Kluckow M, Gill AW, Hooper SB & Polglase GR. (2022). Single versus continuous sustained inflations during chest compressions and physiological-based cord clamping in asystolic lambs. *Arch Dis Child Fetal Neonatal Ed* **107,** 488-494.

Seow BKL, McDougall ARA, Short KL, Wallace MJ, Hooper SB & Cole TJ. (2019). Identification of Betamethasone-Regulated Target Genes and Cell Pathways in Fetal Rat Lung Mesenchymal Fibroblasts. *Endocrinology* **160,** 1868-1884.

Stevens DR, Yeung E, Hinkle SN, Grobman W, Williams A, Ouidir M, Kumar R, Lipsky LM, Rohn MCH, Kanner J, Sherman S, Chen Z & Mendola P. (2023). Maternal asthma in relation to infant size and body composition. *J Allergy Clin Immunol Glob* **2,** 100122.

Süvari L, Helve OM, Kari MA, Turpeinen LU, Palojärvi PA, Leskinen MJ, Andersson S & Janér AC. (2021). Glucocorticoids, sodium transport mediators, and respiratory distress syndrome in preterm infants. *Pediatr Res* **89,** 1253-1260.

Tannenbaum J & Bennett BT. (2015). Russell and Burch's 3Rs then and now: the need for clarity in definition and purpose. *J Am Assoc Lab Anim Sci* **54,** 120-132.

Trotter A, Ebsen M, Kiossis E, Meggle S, Kueppers E, Beyer C, Pohlandt F, Maier L & Thome UH. (2006). Prenatal estrogen and progesterone deprivation impairs alveolar formation and fluid clearance in newborn piglets. *Pediatr Res* **60,** 60-64.

Vidaeff AC, Belfort MA, Kemp MW, Saade GR, Caughey AB, Wapner RJ, Goldenberg RL & Jobe AH. (2023). Updating the balance between benefits and harms of antenatal corticosteroids. *Am J Obstet Gynecol* **228,** 129-132.

Visconti K, Senthamaraikannan P, Kemp MW, Saito M, Kramer BW, Newnham JP, Jobe AH & Kallapur SG. (2018). Extremely preterm fetal sheep lung responses to antenatal steroids and inflammation. *Am J Obstet Gynecol* **218,** 349.e341-349.e310.

Wallace MJ, Hooper SB & Harding R. (1995). Effects of elevated fetal cortisol concentrations on the volume, secretion, and reabsorption of lung liquid. *Am J Physiol Regul Integr Comp Physiol* **269,** R881-887.

Wallace MJ, Hooper SB & Harding R. (1996). Role of the adrenal glands in the maturation of lung liquid secretory mechanisms in fetal sheep. *Am J Physiol Regul Integr Comp Physiol* **270,** R33-40.

Wang G, Murphy VE, Namazy J, Powell H, Schatz M, Chambers C, Attia J & Gibson PG. (2014). The risk of maternal and placental complications in pregnant women with asthma: a systematic review and meta-analysis. *J Matern Fetal Neonatal Med* **27,** 934-942.

Westover AJ, Hooper SB, Wallace MJ & Moss TJ. (2012). Prostaglandins mediate the fetal pulmonary response to intrauterine inflammation. *Am J Physiol Lung Cell Mol Physiol* **302,** L664-678.

Willet KE, Jobe AH, Ikegami M, Polk D, Newnham J, Kohan R, Gurrin L & Sly PD. (1997). Postnatal lung function after prenatal steroid treatment in sheep: effect of gender. *Pediatr Res* **42,** 885-892.

Wooldridge AL, Clifton VL, Moss TJM, Lu H, Jamali M, Agostino S, Muhlhausler BS, Morrison JL, De Matteo R, Wallace MJ, Bischof RJ & Gatford KL. (2019). Maternal allergic asthma during pregnancy alters fetal lung and immune development in sheep: potential mechanisms for programming asthma and allergy. *J Physiol* **597,** 4251-4262.

World Health Organization. (2022). WHO recommendations on antenatal corticosteroids for improving preterm birth outcomes. World Health Organization, Geneva.

Yamaoka S, Crossley KJ, McDougall ARA, Rodgers K, Zahra VA, Moxham A, Te Pas AB, McGillick EV & Hooper SB. (2022). Increased airway liquid volumes at birth impair cardiorespiratory function in preterm and near-term lambs. *J Appl Physiol (1985)* **132,** 1080-1090.

Yland JJ, Bateman BT, Huybrechts KF, Brill G, Schatz MX, Wurst KE & Hernández-Díaz S. (2020). Perinatal outcomes associated with maternal asthma and its severity and control during pregnancy. *J Allergy Clin Immunol Pract* **8,** 1928-1937.e1923.

**Additional information**

**Data availability**

This article has an online data supplement containing further details on methods including induction of the maternal asthma phenotype, ewe lung function measures, histological analyses, real-time PCR and glucocorticoid analyses. The online data supplement also includes additional results on the blood biochemistry of lambs during ventilation and plasma and lung tissue glucocorticoid concentrations. The data generated and analysed during this study have been uploaded as supplementary files or is available from the corresponding author on reasonable request.

**Competing interests**

The authors declare no conflicts of interest.

**Author contributions**

Conception or design of the work: KLG, JLM, MJS, AT, BSM, RJB, VC, TJM and MJW

Experiments were performed by: JLR, AJR, SJH, JRTD, SLH, ASM, CGD, SJ, KLG, MJS, and JLM

Acquisition, analysis or interpretation of data for the work: JLR, SJ, SJH, JRTD, SLH, ASM, MDW, KLG, JLM, MJW, AND and MJS

Drafting the work or revising it critically for important intellectual content: JLR, KLG, JLM, MJW, and MJS

Final approval of the version to be published and agreement to be accountable for all aspects of the work: All.

**Funding**

The sheep experiments were funded by a Channel 7 Children’s Research Foundation grant (20190628) to KLG, JLM, MJS, AT, BSM, RJB, VC, TJM and MW and Robinson Research Institute (Covid-19 impact support funding to KLG). JLM and the molecular studies were funded by an ARC Future Fellowship (Level 3; FT170100431). JLR and SJH are supported by Australian Government Research Training Program Scholarships and Healthy Development Adelaide/Channel 7 Children’s Research Foundation PhD Excellence Supplementary Scholarships. AJR and CGD are supported by Australian Government Research Training Program Scholarships. VLC is supported by a National Health and Medical Research Council Senior Research Fellowship (APP1136100).

**Acknowledgements**

We acknowledge the contribution of members of the Early Origins of Adult Health Research Group in assistance with tissue collection. We acknowledge the technical assistance of the National Imaging Facility, an NCRIS capability, at PIRL, SAHMRI, and their animal technicians. We also acknowledge the Victorian Government’s Operational Infrastructure Support Program.

|  | **Control** | | | **Asthma** | | **Asthma+beta** | | **Significance** | | |
| --- | --- | --- | --- | --- | --- | --- | --- | --- | --- | --- |
|  | **Male** | | **Female** | **Male** | **Female** | **Male** | **Female** | **P_treatment_** | **P_lamb sex_** | **P_interaction_** |
| Number of ewes | 11 | | | 9 | | 8 | |  |  |  |
| Ewe body weight (kg) | 73.3 (6.8) | | | 78.9 (4.6) | | 80.7 (6.7) | |  |  |  |
|  |  |  | |  |  |  |  |  |  |  |
| Number of lambs (numbers per litter size)^2^ | 9 (4 S, 5 T) | 8 (1 S, 7 T) | | 8 (1 S, 7 T) | 6 (3 S, 3 T) | 5 (2 S, 3 T) | 7 (2 S, 5 T) |  |  |  |
| Lamb birth weight (kg) | 4.80 (0.34) | 4.53 (0.78) | | 4.74 (0.69) | 4.54 (0.54) | 4.52 (0.79) | 4.13 (0.75) | 0.461 | 0.205 | 0.974 |
| Lamb birth weight (% of ewe body weight) | 6.56 (0.58) | 6.25 (0.56) | | 5.91 (0.98) | 5.84 (0.88) | 5.40 (0.85) | 5.23 (1.30) | 0.088 | 0.524 | 0.760 |
| Lamb lung weight (g) | 162 (15) | 153 (19) | | 165 (23) | 160 (27) | 148 (36) | 138 (30) | 0.250 | 0.480 | 0.946 |
| Lung weight (% of lamb weight) | 3.3 (0.5) | 3.4 (0.5) | | 3.5 (0.3) | 3.6 (0.3) | 3.3 (0.5) | 3.4 (0.5) | 0.326 | 0.711 | 0.479 |
| Lamb brain weight (g) | 56 (2) | 56 (4) | | 54 (15) | 61 (5) | 58.2 (5) | 58 (4) | 0.666 | 0.320 | 0.314 |
| Lamb brain weight (% of lamb weight) | 0.12 (0.01) | 0.12 (0.01) | | 0.13 (0.03) | 0.14 (0.02) | 0.13 (0.03) | 0.15 (0.03) | 0.085 | 0.254 | 0.922 |

**Table 1. Ewe and lamb weights at delivery^1^**

Data are mean (SD) unless otherwise indicated. Statistical significance (*P* < 0.05) is shown in bold; N/A, not analysed. One control twin died before delivery and was not included. ^1^Lambs were delivered by Caesarean section at 140 days of gestation (term = 150 days). ^2^Litter size is indicated by S = singleton, T = twin. ^3^Although there was an overall effect of treatment, ewe body weight at delivery did not differ between any pairs of treatments.

**Figure Legends**

***Figure 1. Flow diagram with ewe and lamb numbers.*** *Pregnant ewes included in this study are indicated in blue boxes. HDM = house dust mite. HDM-specific IgE = house dust mite-specific immunoglobulin E.*

***Figure 2.*** ***Ventilator parameters during the neonatal lung function study.*** *Data are ventilator settings and output throughout the 45-minute ventilation period: relative tidal volume (A), fraction of inspired oxygen (B, FiO_2_), minute ventilation (C), and dynamic lung compliance (D). Data are from lambs born to control ewes (control, open shapes, male n=8, female n=7) asthmatic ewes (asthma, grey shapes, male n=7, female n=5) and lambs born to asthmatic ewes treated with antenatal betamethasone (asthma+beta, closed shapes, male n=4, female n=7). Data are separated by sex: male lambs (circles, left panels) and female lambs (triangles, right panels). Data were analysed by mixed models, with time as a within-animal factor, and are presented as mean (SD). Statistically significant effects (P < 0.05) are shown in bold and interactions are reported when significant. Bonferroni correction was used to determine differences between groups where a treatment effect was significant.* *^#^controls < asthma+beta. ^asthma* ≠ *control. *asthma < asthma+beta. ^$^male < females.*

***Figure 3. Physiological parameters in lambs during the neonatal lung function study.*** *Data are from lambs born to control ewes (control, open shapes, male n=8, female n=7) asthmatic ewes (asthma, grey shapes, male n=7, female n=5) and lambs born to asthmatic ewes treated with antenatal betamethasone (asthma+beta, closed shapes, male n=4, female n=7). Data show end-tidal carbon dioxide (A), and heart rate (B). Data are separated by sex: male lambs (circles, left panel) and female lambs (triangles, right panel). Data are mean (SD). Statistical significance (P < 0.05) is shown in bold, and interactions are reported when significant. Bonferroni correction was used to determine differences between groups where an overall treatment effect was significant. ^#^controls ≠ asthma+beta. *asthma < asthma+beta. ^†^males > females. ^&^no significant differences after Bonferroni post-hoc tests.*

***Figure 4. Plasma and lung tissue cortisol concentrations.*** *Data shows cortisol concentrations in plasma (A) and lung tissue (B). Data are separated by lamb sex into males (circles) and females (triangles). Data are from* *control lambs (open shapes, male n=8-9, female n = 5-7), lambs born to asthmatic ewes (asthma, grey shapes, male n=8, female n=6), and lambs born to asthmatic ewes treated with antenatal betamethasone (asthma+beta, closed shapes, male n=4-5, female n=6-7). Each symbol indicates data from one lamb, with whisker plots showing the mean ± SD for each group. Statistical significance (P < 0.05) is shown in bold. Bonferroni correction was used to determine differences between groups where an overall treatment effect was significant, indicated by different letters. Letters indicate differences between groups (a, b, P < 0.05). P_int_ = treatment*sex interaction analysis*

***Figure 5. Lamb lung structural analyses and surfactant protein mRNA expression.*** *Representative H&E stained lamb lung sections (A-C, 10x magnification, Hamamatsu NanoZoomer 2.0-HT) and lung sections immunolabelled for surfactant protein B (E-G, 60x magnification, Hamamatsu NanoZoomer 2.0-HT) from control lambs (A, D-E, H, open shapes, male n=8, female n = 7), lambs born to asthmatic ewes (B, D, F, H, asthma, grey shapes, male n=8, female n=4), and lambs born to asthmatic ewes treated with antenatal betamethasone (C-D, G-H, asthma+beta, closed shapes, male n=5, female n=6). Data (D, H-L) are separated by sex: male lambs (circles) and female lambs (triangles). Four lambs were excluded from tissue-to-airspace analyses due to poor fixation of the lung. Type II cell density data points are means of counts from two tissue samples taken from inflation-fixed left lamb lungs. Cells were only classified as type II cells (closed arrows) if they met all three criteria: SP-B positive (brown cells), with a rounded morphology that bulged into alveolar space. SP-B-positive cells that did not meet colour, morphological and location criteria were not counted as type II cells. mRNA expression of SFTPA (panel I), SFTPB (panel J), SFTPC (panel K), and SFTPD (panel L) are relative to three stable housekeeping genes (BACTIN*, *HPRT*, and *TBP). Data points (D, H-L) are means of data from two lung regions for each animal. Data are individual animals (shapes) with error bars showing mean (SD). Statistical significance (P < 0.05) is shown in bold. Bonferroni correction was used to determine differences between groups (P < 0.05), indicated by different letters. P_int_ = treatment*sex interaction analysis.*
